# Multi-scale surface topography to minimize adherence and viability of nosocomial drug-resistant bacteria

**DOI:** 10.1016/j.matdes.2017.11.074

**Published:** 2018-02-15

**Authors:** Jafar Hasan, Shubham Jain, Rinsha Padmarajan, Swathi Purighalla, Vasan K. Sambandamurthy, Kaushik Chatterjee

**Affiliations:** a Department of Materials Engineering, Indian Institute of Science, Bangalore 560012, India; b Mazumdar Shaw Centre for Translational Research, NH Health City, Bangalore 560099, India

**Keywords:** Wet etching, Nanotopography, Biofilm, Antifouling, Bactericidal, Nosocomial infections, Drug resistant bacteria

## Abstract

Toward minimizing bacterial colonization of surfaces, we present a one-step etching technique that renders aluminum alloys with micro- and nano-scale roughness. Such a multi-scale surface topography exhibited enhanced antibacterial effect against a wide range of pathogens. Multi-scale topography of commercially grade pure aluminum killed 97% of *Escherichia coli* and 28% of *Staphylococcus aureus* cells in comparison to 7% and 3%, respectively, on the smooth surfaces. Multi-scale topography on Al 5052 surface was shown to kill 94% of adhered *E*. *coli* cells. The microscale features on the etched Al 1200 alloy were not found to be significantly bactericidal, but shown to decrease the adherence of *S*. *aureus* cells by one-third. The fabrication method is easily scalable for industrial applications. Analysis of roughness parameters determined by atomic force microscopy revealed a set of significant parameters that can yield a highly bactericidal surface; thereby providing the design to make any surface bactericidal irrespective of the method of fabrication. The multi-scale roughness of Al 5052 alloy was also highly bactericidal to nosocomial isolates of *E*. *coli*, *K*. *pneumoniae* and *P*. *aeruginosa*. We envisage the potential application of engineered surfaces with multi-scale topography to minimize the spread of nosocomial infections.

## Highlights

• Induction of multi-scale surface architecture is achieved through wet etching on aluminum substrates.
• The multi-scale surface architecture on Al and its alloys kills as well as repels bacterial cells.
• Multi-drug resistant bacterial strains isolated from the hospitals are also lysed by the etched Al surfaces
• Combination of ranges of surface roughness parameters are identified for designing surfaces with high bactericidal activity.

## Graphical abstract

**Figure:**
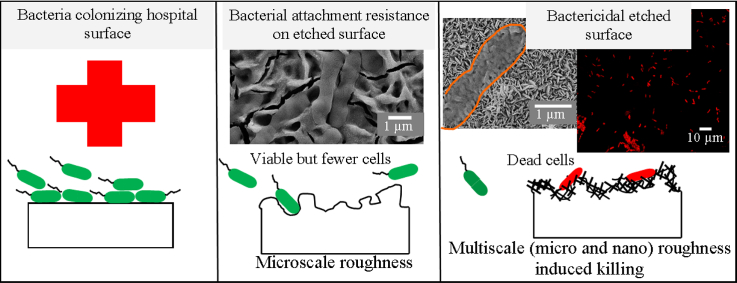

## 1 Introduction

Evolutionary processes over billions of years have led to design optimization to tackle a variety of adverse challenges encountered by living systems. Biological systems offer much inspiration for engineering surfaces with unique functionalities. Several natural surfaces with hierarchical structures of size ranging from the macro-scale to the nano-scale exhibit multiple functions on a single surface. Such surfaces possess superhydrophobic, self-cleaning and/or bacteria-killing properties to maintain an ultra-clean surface despite the abundance of contaminants in their surrounding environment [1], [2], [3], [4]. However, not all kinds of superhydrophobic surfaces exhibit self-cleaning and antifouling behavior [5]. The antifouling activity of a surface is dependent on its ability to resist different kinds of contaminants such as bacteria, yeasts, diatoms, larva or algal spores, see weeds and others which are all of variety of sizes [6], [7], [8]. The antifouling effect of a surface exhibiting micron and nanoscale roughness will only be able to resist only those fouling agents which are in the similar length scales such as bacteria. The multi-scale roughness present on various plant and animal surfaces such as rose petals, lotus leaves, taro leaves, rice leaves, legs of water strider, shark skin, wings of cicadae and dragonfly, gecko feet represent a few of these examples [2], [3], [9], [10], [11].

Shark skin with microscale topography is well known to resist the attachment of bacterial cells [12]. More recently, it was reported that cicada and dragonfly wings can kill several pathogenic bacteria such as *E*. *coli*, *B*. *subtilis and P*. *aeruginosa* [13], [14]. The bactericidal property of their wings is attributed to the mechanical rupture of bacterial cell membrane induced by the physical interaction of cells with the nanotopography present on the wing. By means of this design, nature has evolved effective means to reduce the biofouling of surfaces. Inspired by such natural surfaces, researchers have induced multi-scale surface roughness on several artificial surfaces to geometrically check the behavior of bacterial attachment and delay the formation of biofilms [10], [13], [14], [15], [16], [17], [18]. Surface topography based strategies are gaining popularity as an alternate to chemical approaches [10], [16], [17], [18], [19] or in synergy with chemical approaches to impart antibacterial effect [20]. It is difficult to engineer microscale and nanoscale surface structures using simple techniques that may be easily scaled. Since some of the natural surfaces have periodic topography, bacterial interaction on artificial patterned substrates has now become a subject of growing interest [21], [22], [23]. Nevertheless, the current micro- and nanofabrication methods are expensive, slow and cumbersome. Readers are directed to very detailed reviews on micro- and nanostructured materials with focus on fabrication, characterization and cellular attachment behavior [24], [25], [26].

It is well known that the initial bacterial adherence to surfaces is the critical first step in biofilm formation and all antibacterial surfaces are typically engineered to minimize this first reversible step. Once attached to the surface, bacteria grow to form colonies which eventually leads to the formation of biofilms by entrapping nutrients and other microorganisms [27]. This makes the cells more resistant to antibacterial agents and disinfectants [28], [29]. Biofilm formation on surfaces imposes a daunting challenge with profound implications in several fields including the hospital environment, food packaging, ship hulls, aircraft components, household objects, medical instruments and implants, and industrial pipelines, leading to breach in safety and hygiene as well as material failure [27], [30]. For example, microbial contamination of aircraft fuel tanks and marine structures made of Al alloys is a widespread problem [31], [32].

In a healthcare setting, biofilm formation on medical devices or hospital surfaces could provide an ideal route for the transmission of nosocomial or hospital-acquired infections owing to the difficulty in deep cleaning of contaminated surfaces. Transmission of nosocomial infections among patients sharing the same bed and other common medical equipment can result in life threatening infections due to multidrug resistant (MDR) gram-negative pathogens [33], [34], [35]. Several reports have highlighted the ability of these MDR pathogens to easily spread across the globe and be responsible for 30% of the nosocomial infections and 70% of infections in the intensive care units (ICU) [34], [36]. The advances in modern medicine that employs the use of mechanical ventilators and invasive devices to perform complex surgeries entails the need for repeated use of these devices between surgeries by employing repetitive cleaning with disinfectants before and after the surgery. The current practice of employing broad spectrum chemical disinfectants to sterilize heat sensitive components has been rendered ineffective due to the emergence of drug resistant pathogens. Different strategies to reduce bacterial contamination are being explored. Recently, the effect of micropatterned topography to induce bacteria resistant surfaces in hospital settings has been studied [37]. However, more extensive studies are required to test the effect of topography dependent bacteria-killing surfaces for applications in hospitals. Therefore, there is a critical need to devise novel strategies that employ antibacterial surfaces in the hospitals with the use of bactericidal or bacteria-resistant (antibiofouling) surface coatings such as silver paints, copper or hydrophobic coatings to achieve robust disinfection rates [1], [38], [39]. Other emerging strategies utilize chemical functional groups or grafting of polymer chains that either repel or lyse the bacterial cells through contact killing or leaching. But there are many limitations associated with these approaches [19]. For instance, bacterial resistance is on the rise against chemical disinfectants. Secondly, the leaching rate of the antibacterial agents is either too quick or too slow. Thirdly, the amount of the antibacterial agents added onto the surface is not optimally maintained. Lastly, the surfaces are not durable enough and may require replacements [19], [40], [41], [42], [43], [44]. Moreover, several of these agents can be toxic or carcinogenic during long-term use thereby limiting their usage in applications that involve human contact.

Today, Al alloys have found widespread use in several industries that would immensely benefit from the availability of methods to induce roughness on Al surfaces such as surface coating, anodization and wet etching [45], [46], [47], [48]. Few of these published studies report the generation of superhydrophobic, superoleophobic and self-cleaning Al surfaces prepared by etching [48], [49], [50], [51]. In this study, we aimed to develop a simple etching technique to engender multi-scale topography (microstructures and nanostructures) on Al surface and its alloys to reduce bacterial adherence and viability against nosocomial pathogens. We propose that a combination of microscale and nanoscale features will impart antibiofouling and bactericidal properties, respectively, in a manner observed for several natural surfaces to minimize bacterial attachment, growth and eventually to retard biofilm formation. We also aim to identify a combination of different roughness parameters with values that will render a highly bactericidal surface. To establish a proof of concept, the etched surfaces were tested against several bacterial strains including drug resistant pathogens isolated from a hospital environment.

## 2 Experimental

### 2.1 Surface preparation

All Al samples used here were procured commercially from a local hardware shop and the topography was generated by wet etching technique performed at 25 °C. The controls as well as the etched samples was sterilized by sonicating 10 mL of 70% ethanol followed by 10 mL of 100% of ethanol and 10 mL of 100% methanol solutions. The sonication in each solution was done for 15 min. The Al sheets of 0.5 μm thickness and Al alloys of 2 mm thickness were cut to circular discs of 10 mm diameter using a metal cutter prior to etching. Etching of commercial grade pure Al (cp Al) was performed in 1.0 M sodium hydroxide (NaOH) bath for time intervals of 10, 30 and 60 min. For Al 1200 and Al 5052 alloys, the etching was performed in 0.1 M, 0.5 M and 1.0 M NaOH solution for 10, 30, 60 or 180 min. Etching of Al 5052 was also performed in 1.0 M KOH for 10 and 60 min. The unetched surfaces were used as controls in all the experiments. To test for scalability, larger sheets of 29 cm × 14 cm were prepared and etched, as above. During etching, Al samples were placed at the bottom of the etchant filled vessel placed on a tilt shaker. After etching, the surfaces were immediately sonicated in distilled water and were then rinsed with 250 mL of distilled water.

### 2.2 Surface characterization

The static contact angle of ultrapure water (Sartorius Arium) was measured using a contact angle goniometer (OCA 15EC, Dataphysics) at various surfaces. The image of the water droplet was taken 1 s after dispensing 1 μL of water on the sample. The contact angle was measured using the ImageJ software. Three independent replicates were used for each sample. Surface morphology of the etched surfaces were observed using a scanning electron microscope (SEM, Ultra55, Gemini) set at 7 kV with a secondary or in-lens detector. Optical profilometry was performed using a 3D non-contact optical profiler TalySurf CCI by a 50 × lens.

Surface topography of the top regions of the control and etched aluminum was measured using an atomic force microscope (Bruker, Dimension Icon ScanAsyst) in tapping mode. A cantilever of Tespa model (Bruker) with frequency in the range of 273–377 kHz and spring constant of 20–80 N/m and was utilized. Roughness analysis from the five different AFM images scanned over 10 μm × 10 μm areas on triplicates of each sample type was performed using the freely available Gwyddion software. A total of ten roughness parameters were analyzed, namely, average roughness (R_a_), root mean square roughness (R_q_), average maximum height of the roughness (R_t_), ten-point height (R_z_), ratio of surface area to projected area (S.A./P.A.), autocorrelation length (*T*) measured using one-dimensional autocorrelation function, skewness (R_skw_) and kurtosis (R_kur_) whereas the other two were spatial based, namely, average wavelength (λ_a_) and root mean square wavelength (λ_q_) of the surfaces.

The Al, O and Na content of the surfaces were characterized using X-ray photoelectron spectroscopy (XPS, Axis Ultra). High resolution XPS spectra were recorded using a monochromatic Al source (1.486 keV, Kratos Analytical) at the outermost surfaces. X-ray diffraction (XRD) patterns of the Al alloy samples were characterized using Cu-Kα radiations (PANalytical, X'Pert Pro).

### 2.3 Bacterial response

Bacterial isolates of *Escherichia coli* (ATCC 25922) and *Staphylococcus aureus* (ATCC 25923) were used for the antibacterial studies. Cells were grown in 50 mL of sterile nutrient broth (HiMedia) overnight at 37 °C with shaking at 180 rpm in an orbital shaker. Bacterial cultures were sub-cultured on nutrient agar (HiMedia) to isolate single colonies. The bacterial cells were harvested during the logarithmic phase of growth and the bacterial numbers adjusted to an OD_600_ (optical density at 600 nm) of 0.10 in nutrient broth. Samples of cp Al or the alloys were immersed in 400 μL of bacterial suspension in a sterile 48-well polystyrene (PS) plate. The surfaces were incubated for 4 h at 37 °C before imaging the surface. To further examine the ability of the etched surfaces of the two Al alloys to limit proliferation of *S*. *aureus* cells, the surfaces were incubated for 24 h at 37 °C. After 4 h of incubation at 37 °C, as above, samples were bath-sonicated in sterile phosphate buffered solution (PBS, 1 ×) for 1 min to remove the loosely adhered cells. Thereafter, fresh nutrient broth was added on the surfaces and incubated for an additional 20 h to test viability of the adherent bacterial cells. The cell density was measured using low magnification (4000 ×) SEM images to visualize the bacterial cells. The cells were counted from the images and normalized to the surface area. Cell counting was performed on ten randomly selected areas of three independent replicates from each surface.

To assess the morphology of the adhered bacterial cells, the un-etched (control) and etched surfaces were washed with fresh PBS and the cells were fixed with 2.5% glutaraldehyde for 20 min. Following fixation, the samples were rinsed with PBS solution and sequentially dehydrated in 50%, 60%, 70%, 80%, 90% and 100% of ethanol solution. Then, the samples were sputtered with gold coating prior to imaging using a SEM (Ultra55, Gemini) set at 7 kV with a secondary or in-lens detector.

Viability of the adherent bacterial cells was determined by staining cells stained with the LIVE/DEAD® BacLight™ Bacterial Viability kit (Molecular Probes, Invitrogen). Adherent cells were stained using 3.3 mM SYTO 9 and 20 mM propidium iodide for 15 min and imaged for live (green) and dead cells (red), respectively. Confocal laser scanning microscopy was also performed using Zeiss LSM 880 microscope with a numerical aperture of 1.4 and 63 × oil immersion lens. The fraction of viable and non-viable cells was determined by counting cells stained as both green and red in color from fifteen images of at least three independent replicates.

### 2.4 Measuring the antibacterial effect of metal surfaces on nosocomial pathogens

This study was conducted with bacterial isolates obtained from patients admitted at the Narayana Health City, Bangalore, India. As part of a routine environmental surveillance, the pediatric ward was sampled for environmental contamination of the following surfaces and equipment: sink, tap, and bedside table, bed handrail (including bed) equipment at bedside, medical equipment and hand gel/soap. These surfaces and equipment are frequently touched by the hands of medical and nursing staff and is a likely source of contamination and spread of nosocomial pathogens. Sterile swabs were used for collecting the bacterial flora on surfaces in an area measuring approximately 10 cm × 10 cm. The swabs were moistened in sterile saline and then used to sample the surface. The swabs were streaked on to sterile chocolate agar plates and incubated for 48 h at 37 °C. Each of the bacterial colonies was speciated using the BD Phoenix Automated system (BD Diagnostic Systems, Sparks, MD) and a MALDI-TOF mass spectrometer (Bruker Daltonics, Bremen, Germany) following the manufacturer's guidelines. Bacterial cultures of *E*. *coli*, *K*. *pneumoniae* or *P*. *aeruginosa* were allowed to attach for 4 h on to Al 5052 surfaces etched and the non-etched smooth control, as described above. SEM imaging and fluorescent staining were performed, as described above. Further information on the antimicrobial susceptibility patterns of the bacterial isolates obtained from the hospital is available in the Supporting Information.

A microbial air sampler system (Himedia-LA474) was used for sampling of air by using chocolate agar plates (Himedia) in a designated area in a microbiology lab at Narayana Health City, Bangalore. The microbial air sampler was operated at an air flow-rate of 100 L/min with a sampling time of 10 min. The bacterial burden in the air was enumerated by counting the number of colony forming units (CFUs) per cubic meter (CFU/m^3^) of air. The bacterial load in air was measured once before each of the test conditions and after exposure of un-etched and etched surfaces (*n* = 7) for one hour. Control run without any Al samples was also performed.

Samples were also collected from five areas post-exposure to room air to evaluate viable bacterial counts on the surface. Moistened sterile cotton swabs were used to evenly sample designated areas measuring 5 cm × 5 cm. All swabs taken from different sites were inoculated on to chocolate agar and culture plates were incubated at 37 °C for 24 h. The number of colonies was recorded as colony-forming units (CFUs)/100 cm^2^.

### 2.5 Statistical analysis

Statistically significant differences between the control and etched surfaces or different conditions were analyzed by 1-way ANOVA (analysis of variance) with Tukey's test for multiple comparisons. Differences were considered statistically significant for *p* < 0.05 and indicated as various symbols in the figures. Non-parametric regression analysis was performed to model the relationship between different roughness parameters and bactericidal activity of the surfaces. The regression was done using locally weighted regression and smoothing scatter (LOWESS) plots in XLSTAT software.

## 3 Results

### 3.1 Effect of NaOH etching on surface architecture of Al and its alloys

**Fig. 1:**
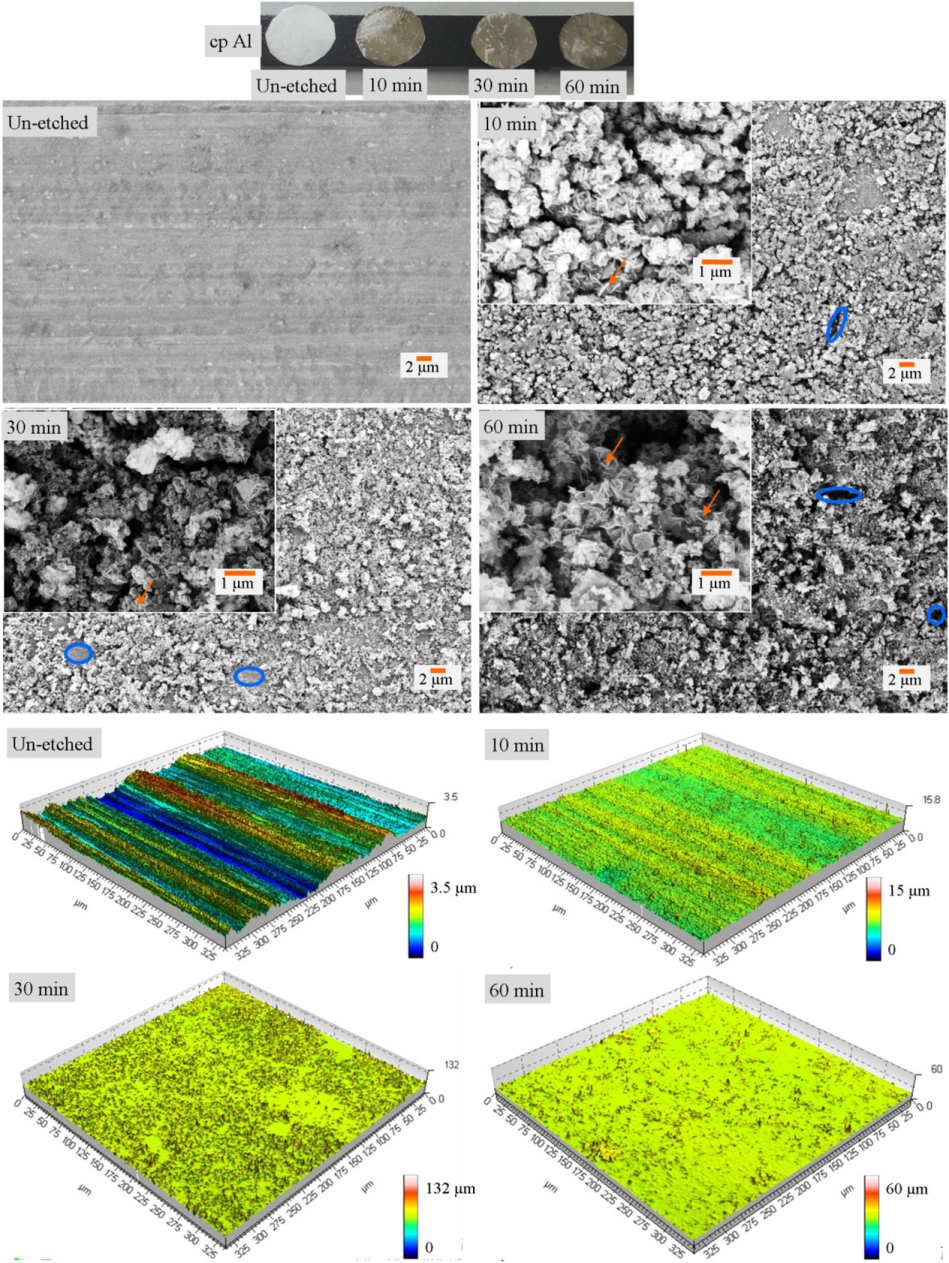
Photographs of the un-etched, 10 min, 30 min and 60 min cp Al samples. Scanning electron micrographs and optical profilometer images of un-etched, 10 min, 30 min and 60 min etched cp Al surfaces. Scale bars are same for all microscopy images.

Wet etching was utilized in this work wherein NaOH was used as the etchant. The etching process is rapid as the micro- and nanostructures are formed within 10 min (Fig. 1). The micro- and nanostructures, respectively, are shown as blue circles and orange arrows, respectively, in Fig. 1B, C, D and E. The optical profilometry and AFM data (Fig. 1 and Supporting Information, Fig. S1) confirm the enhanced multi-scale roughness induced after etching of the cp Al surfaces. However, there is no discernable trend of increasing roughness with etching time.

**Fig. 2:**
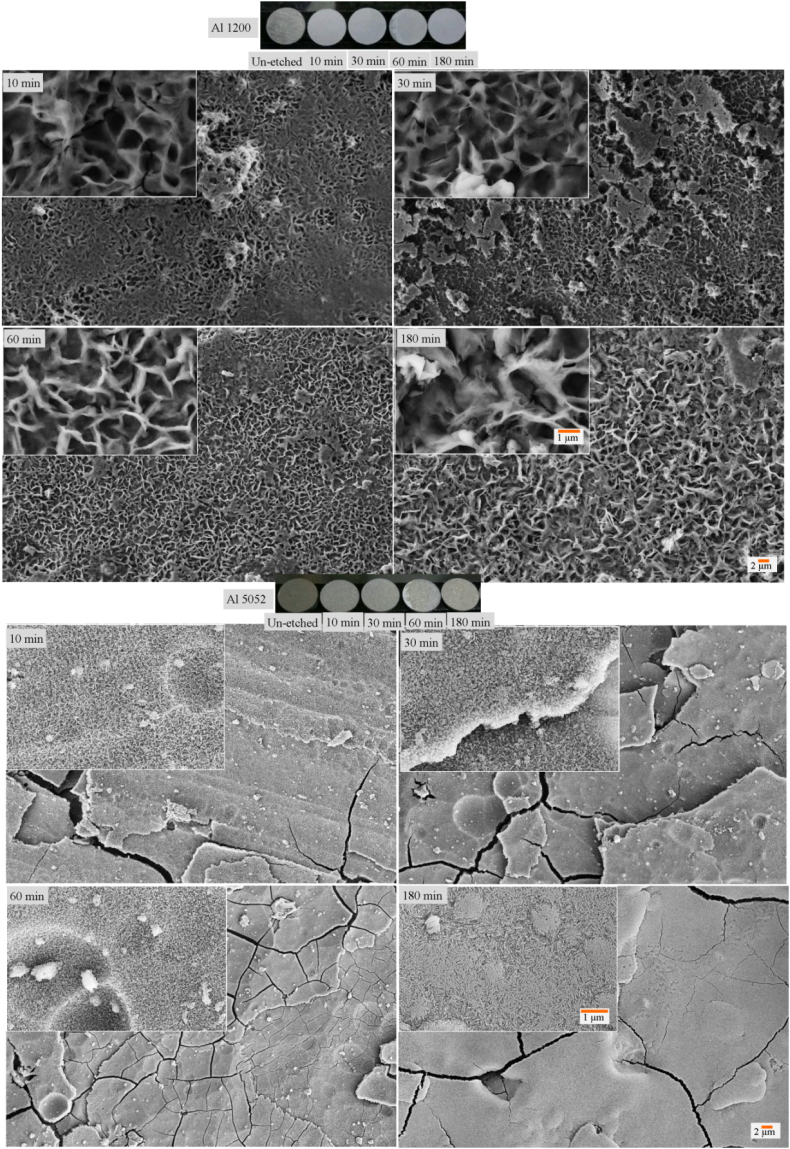
Photographs of Al 1200 alloy etched at different time intervals and corresponding SEM of 10 min, 30 min, 60 min and 180 min etched surfaces. Photographs of Al 5052 alloy etched at different time intervals and corresponding SEM of 10 min, 30 min, 60 min and 180 min etched surfaces.

Etching of Al 1200 and Al 5052 alloys was also performed using NaOH. The surface morphology and topography analysis is seen to generate some microstructures on Al 1200 upon etching (Fig. 2 and Fig. S2).

### 3.2 Scalability of the fabrication of Al surfaces with multi-scale topography

The etching method is scalable and not limited to the small sample dimensions and solution volume mentioned here. Etching was also performed on larger Al sheets (29 cm × 14 cm) for 30 min. A small piece of Al alloys were cut and imaged under SEM and the surface features were found to be same as presented on small 10 mm disc samples that have been used for bacterial studies (Fig. S3).

### 3.3 Surface chemistry analysis

The basic reactions of Al with NaOH are well established. The sodium hydroxide and Al react to produce sodium aluminate and hydrogen. At this high pH, the sodium aluminate form free sodium hydroxide and Al hydroxide. The XPS data revealed that the etched surfaces of the cp Al, Al 1200 and 5052 alloys showed peaks of Na1s which were not present in the control surfaces (SI, Fig. S4). This is due to the sodium hydroxide etching. The XPS spectra of the surfaces after etching revealed the presence of metallic Al as well as its oxide form, which is confirmed by the Al2p and O1s peaks. Note that the presence of the small quantities of alloying elements in the different Al alloys used here seemed to profoundly affect the reactions leading to marked differences in the surface features formed. XRD patterns confirmed the presence of Al on the control as well as etched Al 1200 and 5052 alloy surfaces. There was no change detected in XRD patterns on the etched surfaces (SI, Fig. S5) as the volume of the etched material is limited to the surface.

### 3.4 Surface wettability analysis

The wettability analysis reveals that the cp Al surfaces are hydrophobic and the hydrophobicity is reduced upon etching (SI, Fig. S6). Enhanced wettability is likely due to the change in surface chemistry induced by chemical etching or due to altered topography such that the water droplet may be seeping in to the superficial cracks.

### 3.5 Effect of multi-scale topography on bacterial adherence and viability

**Fig. 3:**
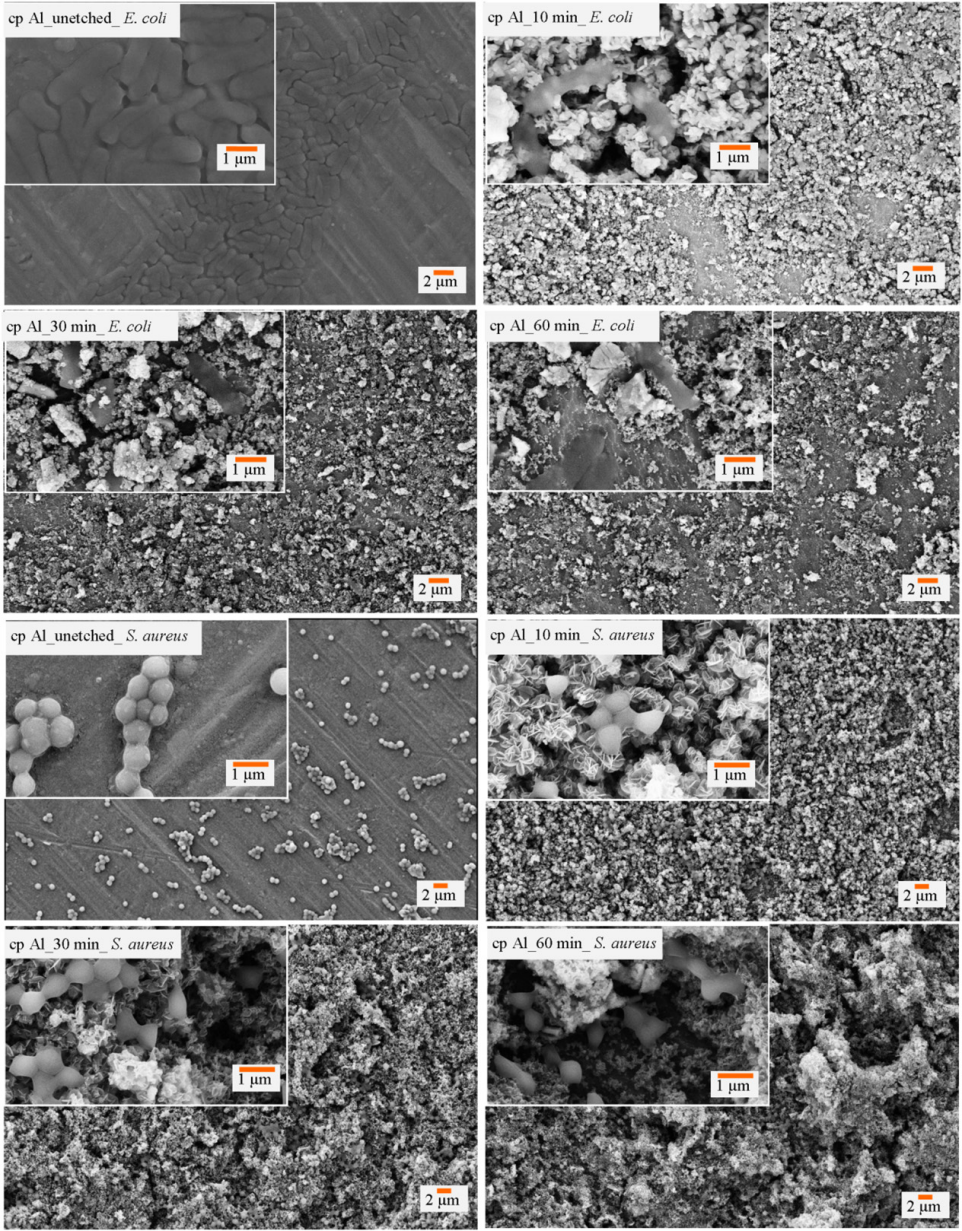
Scanning micrographs depicting the attachment patterns of *E*. *coli* cells on un-etched, 10 min, 30 min and 60 min etched Al surfaces and *S*. *aureus* bacteria on un-etched, 10 min, 30 min and 60 min etched cp Al surfaces.

Adherence of *E*. *coli* cells studied using a SEM and fluorescence microscopy revealed that the micro- and nanostructures formed on the etched cp Al surfaces were effective in lysing the bacterial cells (Fig. 3). The adherence pattern of *S*. *aureus* cells was observed to be affected by the multi-scale roughness (Fig. 3). However, the coccoid shaped cells were not seen to be lysed as much as rod-shaped *E*. *coli* cells. Upon quantification of live and dead cells using fluorescent signals, it was observed that nearly 97% of the attached *E*. *coli* cells were lysed on the 30 minute etched cp Al surface. Similarly, 28% of the attached *S*. *aureus* cells were killed on the etched cp Al surfaces in sharp contrast to 3% on the smooth control surfaces (SI, Fig. S7).

**Fig. 4:**
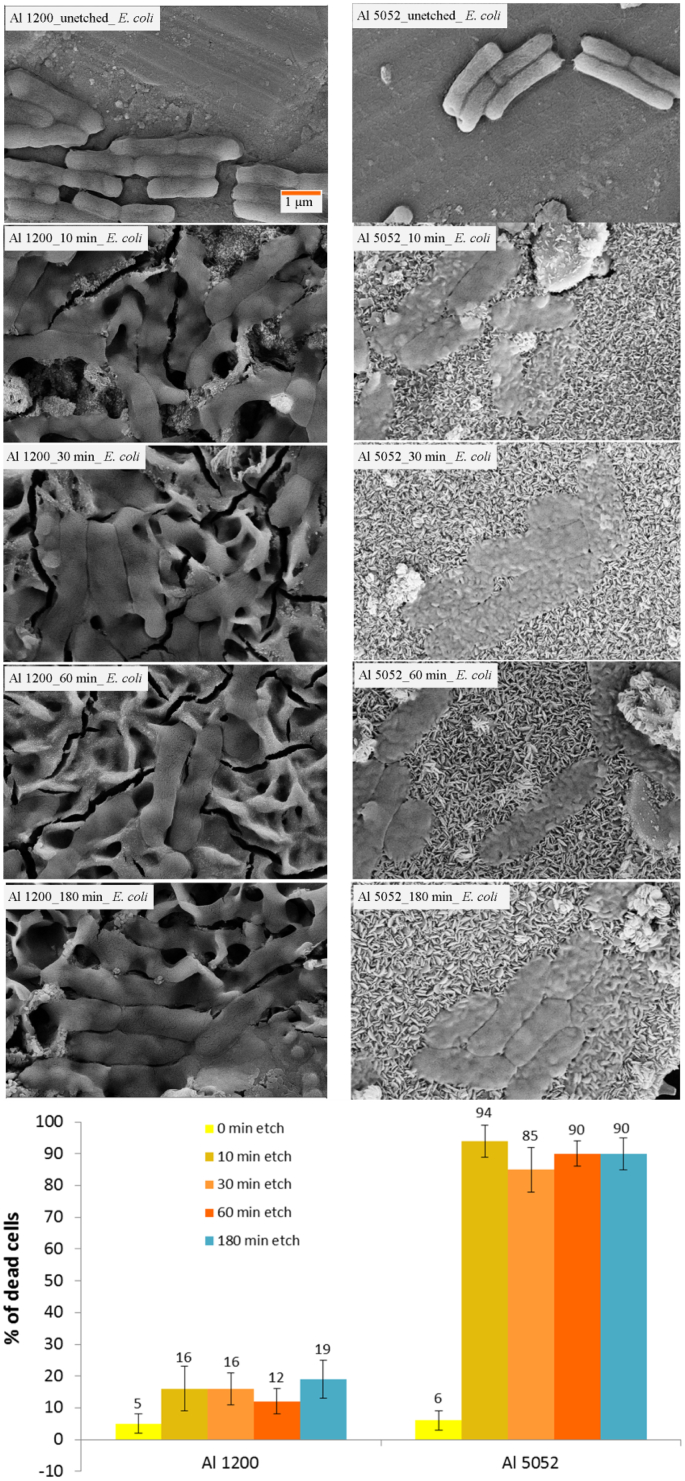
SEM images of bacterial attachment of *E*. *coli* cells on un-etched, 10 min, 30 min, 60 min and 180 min etched Al 1200 (left) and Al 5052 (right) alloy surfaces respectively. The percentage of dead cells attached on each of the surfaces is also presented (bottom).

**Fig. 5:**
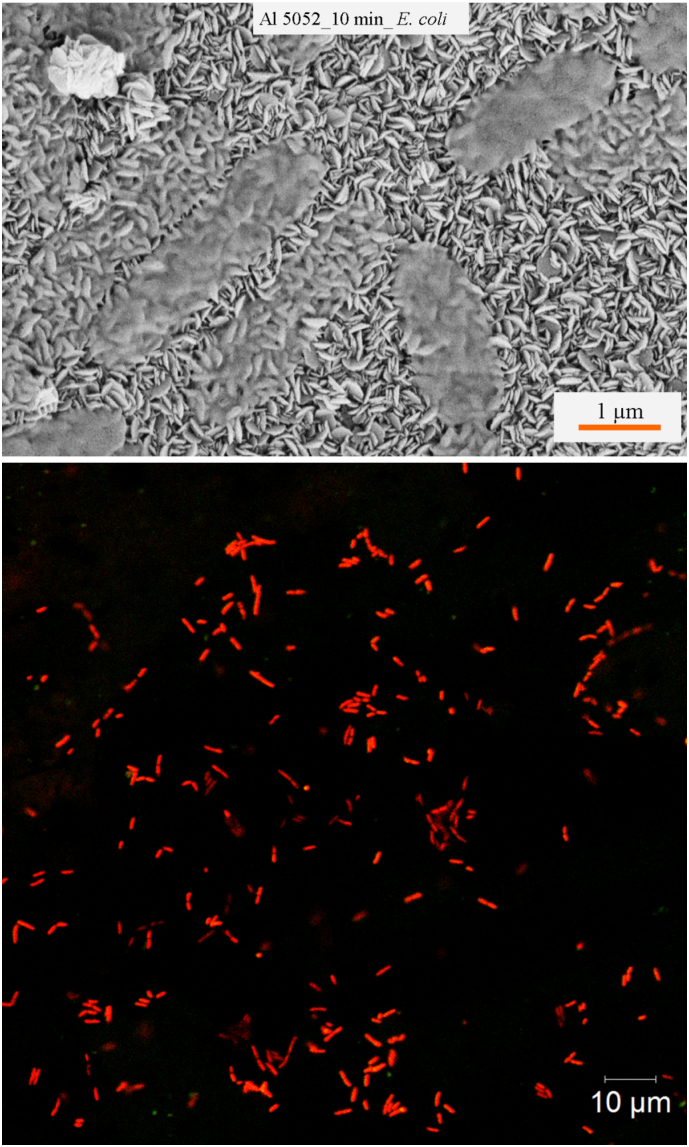
SEM and confocal laser scanning microscopy image of *E*. *coli* cells attached on the 10 min etched Al 5052 alloy surfaces.

Due to the formation of only microscale roughness on the etched surface of Al 1200 alloy, the bacterial attachment shows low killing rate against *E*. *coli* cells (Fig. 4). Also, some damage and distortion to the bacterial cell morphology due to the presence of microscale roughness was observed. Nearly 18% of *E*. *coli* cells were found to be killed due to the micro-featured surface roughness. Also, fewer attached *E*. *coli* cells were observed on the etched surfaces than on the control surfaces. Study of the attachment of coccoid shaped *S*. *aureus* cells on Al 1200 alloy surface revealed that the surface is unable to kill the cells, however, the microscale roughness was found to limit the bacterial growth (SI, Fig. S8). On the etched Al 5052 surfaces, it was observed that 94% of *E*. *coli* cells were non-viable due to membrane lysis induced by the nanostructures present on surfaces (Fig. 4). The multi-scale roughness of the Al 5052 alloy could not kill the spherical shaped, gram-positive cocci *S*. *aureus* (SI, Fig. S9). It appears that the nanoscale features are lysing the cells as can be seen in the scanning electron micrographs (Fig. 5). A representative fluorescent image at high resolution from confocal microscopy of *E*. *coli* cells attached on the etched Al 5052 alloy surface confirms the enhanced bactericidal activity due to the nanofeatures (Fig. 5).

**Fig. 6:**
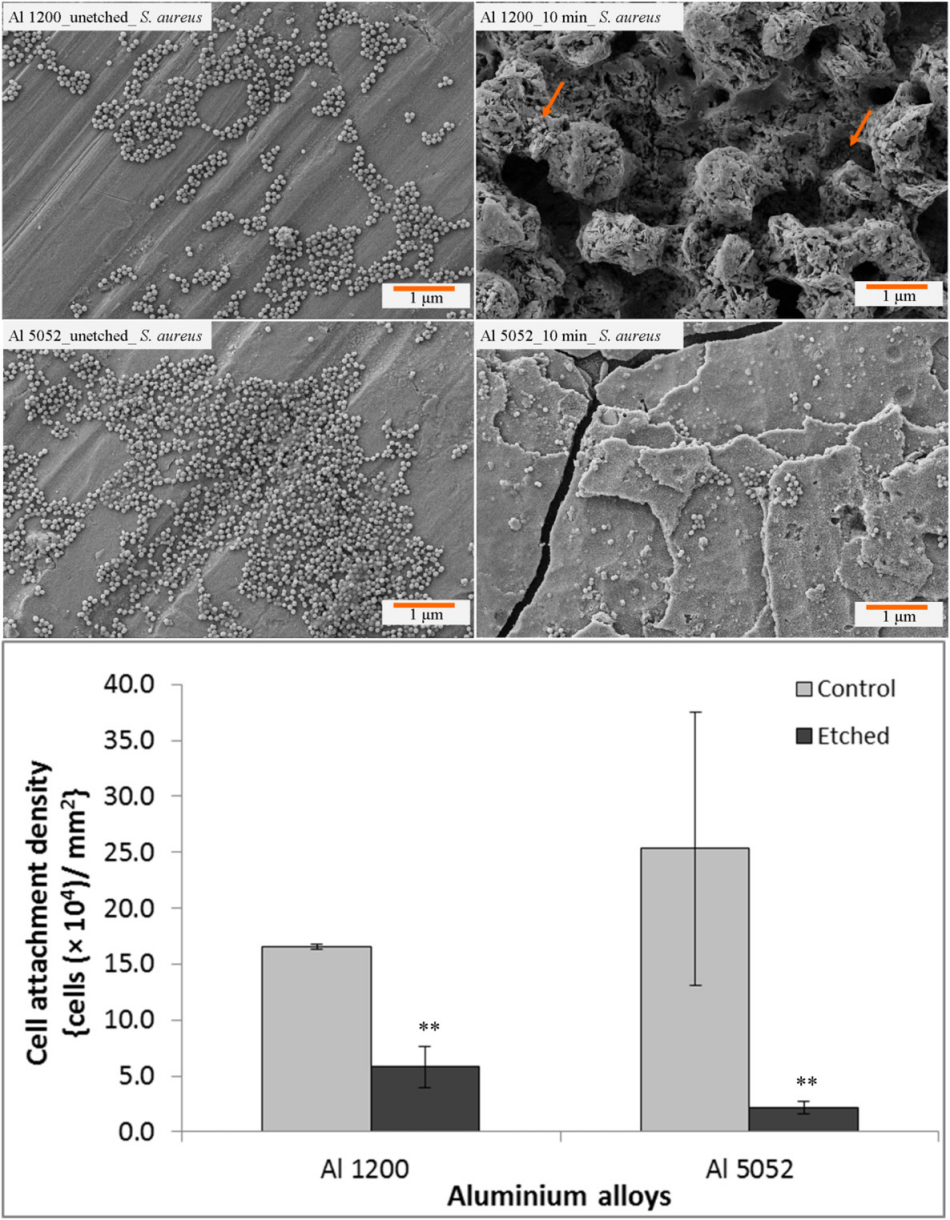
SEM images exhibiting attachment pattern of *S*. *aureus* cells incubated for 24 h on un-etched Al 1200 10 min etched Al 1200, un-etched Al 5052 and 10 min etched Al 5052 alloy surfaces. The attachment density of *S*. *aureus* cells is presented on the control and etched Al alloys (bottom). Statistically significant differences (*p* < 0.01) compared to control Al 1200 alloys and control Al 5052 alloys are indicated by the symbol **.

Interestingly, the number of attached *S*. *aureus* cells was significantly decreased to one-third on the etched Al 1200 alloy surface (Fig. 6) compared to the un-etched control surface. On the etched Al 5052 alloy, the number of adherent cells was significantly reduced to one-tenth (Fig. 6).

Since etched Al 5052 alloy was bactericidal, we proceeded with further tests on the 10 minute etched surface. 10 and 30 minute etching of Al 5052 alloy with weaker base of 0.1 M did not exhibit significant bactericidal activity (~ 25% killing) against *E*. *coli* cells when compared with other surfaces in this study (SI, Fig. S10). However, when the time was increased to 180 min using 0.1 M etchant, the bactericidal activity was comparable to that of the surfaces etched with 1.0 M solution (Table 1). On the other hand, sufficient roughness was generated on the surfaces when we increased the etchant molarity to 0.5 M for time intervals of 10 and 180 min. This led to a marked killing of 94% of *E*. *coli* cells on 10 and 180 minute etched Al 5052 surfaces (Table 1). Similarly, etching the Al 5052 surface with 1.0 M KOH for 10 and 60 min also generated micro-scale roughness that in turn induced bactericidal activity of 46% and 78%, respectively (Table 1). Thus, the presence of the multi-scale roughness imparts the antibacterial activity and it is essentially independent of the etchant used for fabrication.

**Table 1:** Roughness analysis of etched surfaces^a^.

Sample type	R_a_ (nm)	R_q_ (nm)	R_t_ (nm)	R_z_ (nm)	S.A./P.A.	R_skw_	R_kur_	λ_a_ (μm)	λ_q_ (μm)	*T* (μm)	Bactericidal activity against *E*. *coli* (%)
Control Al 5052	20.4 ± 8.1	25.7 ± 9.5	86.8 ± 35.7	41.8 ± 18.7	1.0 ± 0.01	0.5 ± 0.4	− 0.2 ± 0.5	0.3 ± 0.1	0.3 ± 0.1	2.7 ± 1.8	6 ± 2
0.1 M NaOH 10 min Al 5052	23.2 ± 4.9	29.3 ± 5.8	106.8 ± 31.8	52.2 ± 22.9	1.1 ± 0.06	0.1 ± 0.8	0.1 ± 0.6	0.2 ± 0.1	0.2 ± 0.1	2.6 ± 0.9	22 ± 3
0.1 M NaOH 30 min Al 5052	36.7 ± 8.2	40.5 ± 9.8	155.9 ± 38.7	72.8 ± 17.4	1.2 ± 0.21	− 0.4 ± 0.2	− 0.6 ± 0.4	0.3 ± 0.1	0.3 ± 0.1	2.9 ± 1.9	32 ± 8
0.1 M NaOH 180 min Al 5052	127.6 ± 17.1	158.3 ± 17.0	569.4 ± 60.4	230.6 ± 61.5	1.3 ± 0.09	1.3 ± 5.6	− 0.5 ± 0.4	3.1 ± 0.4	2.7 ± 0.5	2.7 ± 0.8	97 ± 2
0.5 M NaOH 10 min Al 5052	96.8 ± 28.8	121.8 ± 36.7	425.5 ± 130.9	209.0 ± 63.6	1.3 ± 0.05	1.8 ± 8.8	− 0.7 ± 0.3	0.3 ± 0.1	0.3 ± 0.1	3.4 ± 0.7	94 ± 5
0.5 M NaOH 180 min Al 5052	135.8 ± 38.6	162.4 ± 40.4	570.0 ± 120.6	256.6 ± 67.8	1.3 ± 0.20	0.3 ± 0.4	− 0.8 ± 0.4	0.3 ± 0.1	0.3 ± 0.1	1.3 ± 0.3	96 ± 4
1 M NaOH 10 min Al 5052	154.0 ± 80.5	189.0 ± 92.4	656.9 ± 293.7	287.9 ± 128.2	1.2 ± 0.17	1.8 ± 6.1	− 0.4 ± 1.0	2.9 ± 0.7	2.7 ± 0.8	1.3 ± 0.3	94 ± 5
1 M NaOH 30 min Al 5052	82.1 ± 38.8	113.9 ± 64.5	307.9 ± 155.9	220.0 ± 96.0	1.2 ± 0.10	0.3 ± 0.5	− 0.1 ± 0.7	2.8 ± 0.9	2.5 ± 0.7	1.5 ± 1.1	85 ± 7
Control Al 1200	27.8 ± 9.8	37.1 ± 13.6	128.2 ± 49.4	53.2 ± 19.9	1.0 ± 0.01	0.3 ± 0.4	− 0.1 ± 0.6	0.4 ± 0.1	0.4 ± 0.1	1.9 ± 0.9	5 ± 3
1 M NaOH 10 min Al 1200	319.6 ± 128.5	387.9 ± 142.8	1282.2 ± 494.7	550.1 ± 212.5	1.6 ± 0.20	0.1 ± 0.3	− 0.8 ± 0.4	0.4 ± 0.1	0.4 ± 0.1	3.5 ± 1.9	16 ± 7
1 M NaOH 60 min Al 1200	607.6 ± 230.4	775.0 ± 314.0	2647.4 ± 933.2	1180.9 ± 379.9	2.1 ± 0.01	0.3 ± 0.4	− 0.5 ± 0.5	0.4 ± 0.1	0.3 ± 0.1	2.2 ± 0.5	12 ± 4
1 M KOH 10 min Al 5052	176.3 ± 58.5	219.7 ± 68.7	705.1 ± 143.8	314.8 ± 71.4	1.2 ± 0.03	− 0.3 ± 0.2	− 0.8 ± 0.3	0.2 ± 0.1	0.2 ± 0.1	2.9 ± 0.4	78 ± 10
1 M KOH 60 min Al 5052	304.8 ± 131.2	396.8 ± 159.5	1368.0 ± 419.0	545.6 ± 158.5	1.7 ± 0.20	0.2 ± 0.3	− 0.2 ± 0.5	0.2 ± 0.1	0.2 ± 0.1	0.8 ± 0.3	46 ± 12
Black Ti 5 min [17]	72.0 ± 20.2	92.1 ± 26.2	416.1 ± 105.6	249.0 ± 74.1	1.4 ± 0.09	0.1 ± 0.1	− 0.1 ± 0.4	0.4 ± 0.1	0.4 ± 0.1	0.8 ± 0.3	78 ± 11
Black Ti 10 min [17]	185.6 ± 20.0	232.5 ± 28.2	903.5 ± 101.9	471.7 ± 73.5	3.5 ± 0.60	0.1 ± 0.1	− 0.6 ± 0.2	0.4 ± 0.1	0.4 ± 0.1	0.3 ± 0.2	95 ± 5

a AFM scans of 10 μm × 10 μm, all data are mean ± S.D.

To further probe the possible influence, if any, of surface properties apart from topography on the bactericidal behavior of the etched Al surfaces, we sputtered 12–15 nm thick layer of gold on the etched Al 5052 surface that induced minimal additional changes in the surface topography.

To examine the effect on biofilm formation, suspension of *E*. *coli* cells were allowed to attach and grow for 2 days on the control and etched Al 5052 alloy substrates. It was found that the cells are able to colonize the control surface whereas the etched surface was successfully able to retard the biofilm formation due to its combinatorial bactericidal and antifouling activities which are generated due to the multi-scale topography (SI, Fig. S12).

### 3.6 Influence of etched surface against hospital derived drug resistant bacterial strains

**Fig. 7:**
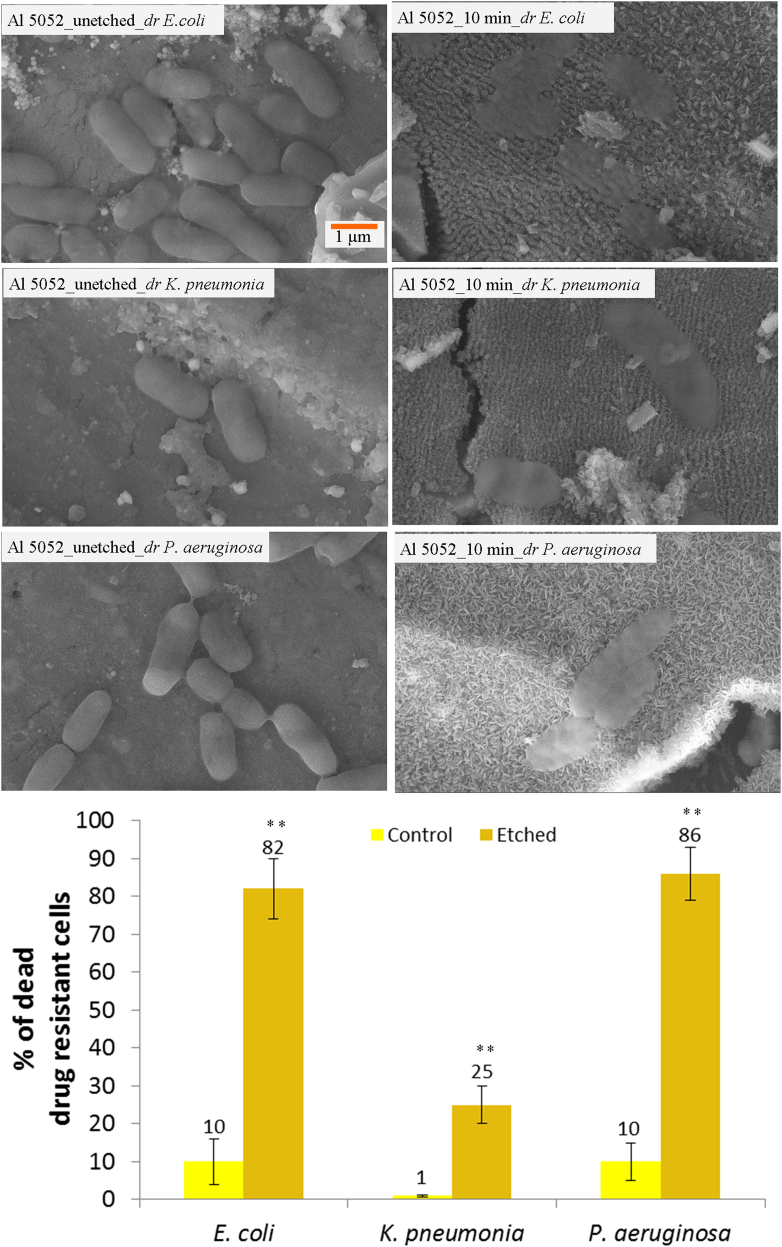
Investigation of the drug resistant (dr) bacterial cells isolated from the hospital environment and attached on the 10 min etched Al 5052 alloy. SEM images of *dr E*. *coli* cells on the control and etched surface; *dr K*. *pneumoniae* cells on the control and etched surface; *dr P*. *aeruginosa* cells on the control and etched surface are shown. Viability analysis of the drug resistant bacterial cells on the control and etched surfaces (bottom). Statistically significant differences (p < 0.01) compared to the control Al 5052 alloy are indicated by the symbol **.

We further explored the antibacterial properties on the 10 min etched Al 5052 alloy surface using drug resistant strains isolated from a hospital environment, namely *E*. *coli*, *K*. *pneumoniae* and *P*. *aeruginosa*. These strains were isolated from patients with nosocomial infections. The SEM images of bacterial attachment on etched Al 5052 alloy showed that the surface roughness is effective in reducing the growth of bacteria found in hospital environment (Fig. 7). The cell appears flat with disrupted morphology on the etched surface in comparison to the characteristic healthy morphology on the control surface. Assessment of cell viability showed that 82% ± 8% of *E*. *coli*, 25% ± 5% of *K*. *pneumoniae*, and 86% ± 7% of *P*. *aeruginosa* cells were lysed by the multi-scale structures of the Al alloy (Fig. 7). Such high bactericidal effect against drug resistant pathogens from the hospital environment mediated by topography alone has not been reported earlier. The etched Al 5052 alloy surface was effectively antifouling as well for all the three hospital-derived strains, as the killing significantly reduced the cell numbers compared to the control surface.

**Fig. 8:**
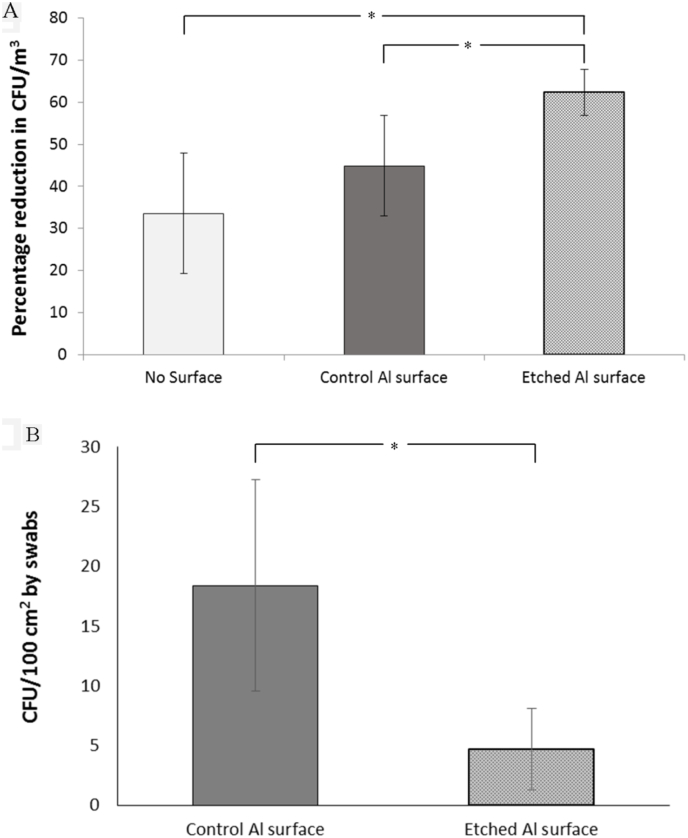
(A) The colony forming units per cubic meter of air in the closed environments measured as percentage reduction after exposure and before exposure for each condition in the presence of no surface, control surfaces and etched surfaces. (B) Colony forming units measured per unit area of 100 cm^2^ by swabbing over several areas from the control and etched sheets. Statistically significant differences (*p* < 0.05) are indicated by the symbol *.

Toward simulating the ability of such etched surfaces in minimizing nosocomial infections, the microbial load on the control and etched Al 5052 alloys was measured using an air sampler in the hospital environment. The air contamination load after the exposure of etched Al 5052 alloy sheets was significantly reduced in comparison to the load after exposure of the control sheets or in the absence of Al sheets (Fig. 8). Similarly, there was a significant reduction in the colonies (18 ± 8 CFU/100 cm^2^) swabbed from the surface of the etched alloys than the colonies (4 ± 3 CFU/100 cm^2^) swabbed from the unetched control surfaces. The antibacterial property of the etched Al 5052 alloy sheets, thus, indicates that such materials could be used to reduce bacterial colonization and potentially help in reducing nosocomial infection rates.

## 4 Discussion

Aluminum and its alloys were the focus of this study because they find widespread use due to their inherent advantages such as low weight, longer life, environmental impact and resistance to corrosion. The use of Al and its alloys can be found in numerous industries. In hospitals, steels, copper alloys and aluminum alloys are some of the commonly used metals used to fabricate objects such as bed rails, stretchers, furniture, doors, toilets, cabinets and ventilators, etc. For example, high pressure laminate aluminum alloys have been used in door frames, profiles and ventilator grids of hospitals. Alloys of Al 5052 and Al1200 were employed in this study because of their wide-ranging usage in areas such as cabinets, fencing, construction, roofing, furniture, containers for food, ship building and aircraft tubing. Differences between the alloys are presented in Supporting Information, SI Table S2.

Interestingly upon etching, there is no nanoscale roughness on Al 1200 as induced on the cp Al surfaces. On the other hand, the surface morphology and topography analysis of the etched Al 5052 shows that both micro- as well as nanoscale features are produced upon etching (Fig. 2 and Fig. S2). However, the multi-scale morphology and architecture on Al 5052 differs from the features present on the cp Al surface. The multi-scale roughness formation on the surfaces of Al and its alloys are similar to that found on the lotus leaves or gecko skin surfaces [15], [38]. The roughness found on natural surfaces is more ordered and regular whereas the micro- and nanostructures on Al substrates are random.

The ease of scalability of the multi-scale roughness shows that the surface preparation can be undertaken on large scale, at low-cost and rapidly when compared with the current techniques to produce hierarchical surfaces [52], [53] or with the other fabrication techniques proposed to prepare nanoscale bactericidal surfaces [13], [17]. The high-throughput etching procedure to create hierarchical roughness is thus unique and scalable for industry.

Oxides of Al are not known for their intrinsic bactericidal activity [54], thereby suggesting that the observed bacterial killing activity cannot be attributed to the chemical composition of the surface. The observed bacterial killing property arises from the nanostructures, which is consistent with the previously reported data on other kinds of surfaces such as insect wings as well as black silicon and black titanium [13], [14], [17]. The nanoscale features are believed to disrupt the bacterial membranes, thereby resulting in bacterial cell lysis and death. In this study, the nanostructures (etched cp Al and etched Al 5052) are observed to be specifically rupturing the cell membrane, while the microstructures offer geographical limitation to bacterial adherence.

The enhanced reduction in the proliferation of *S*. *aureus* cells on the Al 5052 alloy is a result of surface roughness at multi-scales. Surface roughness is known to affect the adhesion force of bacterial cells, which in turn may have caused the reduction of cell attachment on the etched surfaces [55]. Therefore, both of the alloy surfaces have excellent ability to resist bacterial attachment that can be expected to be effective in retarding biofilm formation. These data (Fig. 6) reveal that the multi-scale roughness present on the surfaces is able to create an antifouling mechanism against the coccoid cells.

Despite the altered surface chemistry, the etched surface continued to show excellent bactericidal activity (SI, Fig. S11) demonstrating that the antibacterial phenomenon can be ascribed to the multi-scale surface topography. Other studies have utilized a similar strategy of coating with non-bactericidal gold to demonstrate the topography-mediated bactericidal activity of cicada wings, dragonfly wings and black silicon [13], [14].

Surface roughness plays a significant factor in determining the antibacterial behavior of a given surface. We analyzed ten different roughness parameters of control and etched Al 5052, Al 1200 alloy surfaces with varying etching time, etchant and molarity and also compared with the value of recently reported black titanium surfaces [17] (Table 1). The average roughness (R_a_) values between 80 nm and 300 nm and root mean square roughness (R_q_) values between 110 nm and 380 nm appeared to kill a large fraction (> 85%) of the cells that had attached onto the various surfaces (Table 1). Average roughness (R_a_) and root mean square roughness (R_q_) values outside this range do not seem to be as effective. The average maximum height of the roughness (R_t_) refers to the range of maximum and minimum of the roughness. On analysis, it was observed that values of R_t_ between 300 nm and 900 nm are highly bactericidal. Values of R_t_ higher than 1200 nm may or may not have a detrimental effect on the viability of cells, possibly because it is beyond the height range which can disturb the membrane integrity of *E*. *coli* cells, but higher value can prevent cellular attachment. Ten–point-height (R_z_) is the average of the five highest peaks and five lowest valleys. The value of R_z_ between 200 nm and 500 nm appear to be highly bactericidal, whereas values out of this range do not have effective bactericidal activity. Ratio of the surface area to the projected area of the surfaces showed that values between 1.2 and 1.5 have high bactericidal activity. Although, a very high ratio of 3.5 and higher also cause bactericidal activity as observed for black titanium surface. It appears that ratio above 1.6 and up to 3.5 will not be bactericidal.

Skewness (R_skw_) and kurtosis (R_kur_) are known to be critical parameters in order to control bacterial adhesion [56]. Yet, no significant correlation was apparent here between the bactericidal activity and R_skw_ or R_kur_ of the different surfaces. Smaller values of autocorrelation length (*T*) represent more random surface, whereas high values of *T* represent a more ordered surface architecture [57]. The values of *T* did not seem to have a significant effect on the killing of bacterial cells, but it appears that higher etching time and higher molarity causes more randomness on various surfaces. This is shown by the relatively shorter autocorrelation lengths present on surfaces etched with higher molarity and increased etching time. The spatial parameters, average wavelength (λ_a_) and root mean square wavelength (λ_q_), represent the spacing behavior between the local peaks and the local valleys considering the relative amplitudes and individual spatial frequencies of the surface. It was observed that both of these spatial parameters also do not have any discernable effect on bactericidal activity of the surface.

The multi-scale roughness generated on the etched surfaces of cp Al and its alloys imparts varying effects on bacterial viability. Therefore, it is important to redefine and conceptualize the influence of surface topography on antibacterial behavior. To determine the influence of topography, different kinds of roughness parameters from micro to nanoscale surface topographical features have been recognized to be essential for control of bacterial adherence on surfaces [18], [57]. However, a detailed correlation between several such amplitude- and spatial-based roughness parameters with bactericidal activity has not been reported earlier. It appears that a combination of R_a_, R_q_, R_t_, R_z_ and S.A./P.A. with specified roughness values will likely yield a highly bactericidal surface (Table 1). It has been shown that smaller and/or larger values outside the specified ranges of these roughness parameters will not always yield a bactericidal surface; however, it may yield an antibiofouling surface. Larger values of the above five parameters of roughness may go into the sub-micron and micron range, where the bacterial cell may find some contours to adhere and divide without much resistance from the surface. Similarly, smaller values may also be ineffective to the bacterial cell as they maybe too small in nanometer range of the different roughness parameters. To quantify the observations above, we performed a non-parametric regression analysis. It was confirmed that only R_a_, R_q_, R_t_, R_z_ and S.A./P.A. but not the other parameters have a distinct correlation with the bactericidal activity (SI, Fig. S13A–E) corroborating the qualitative observations described above. It can be seen in AFM images that as the concentration and time of the etching of a particular surface are increasing, the surfaces appear rougher (SI, Fig. S14). To confirm the increased roughness, the quantitative increase in the values of R_a_, R_q_, R_t_, R_z_ and S.A./P.A. can be seen in Table 1. Other roughness parameters did not follow a trend.

Our results are in good agreement with the broad understanding in this field. It is now well established that microscale roughness resists bacterial attachment as the length scales of the surface topography and the bacterial size are of the same order thereby imparting antibiofouling characteristics. Furthermore, when the roughness of the surface is at sub-micron or nanometer length scales, then the viability of the bacterial cells may be affected provided the nanostructured topography disrupts the contact point between cell membrane and the surface [14]. The observations are similar to that on natural surfaces with multi-scale roughness such as taro leaves, lotus leaves and gecko skin [15], [38], [58]. However, the parameters of the topography vary significantly among the various natural surfaces and the etched Al surfaces.

There are numerous reports that show the adhesion behavior of bacterial cells is hindered by the hierarchical roughness of the surfaces [59], [60]. However, the topography induced killing activity against bacterial cells is not present on all kinds of hierarchical surfaces, such as the antifouling surfaces of taro and lotus leaves. Independent of the micron scale roughness, the layout of the nanostructures is critical to damage the bacterial membrane. However, it is not necessary that all kinds of surface nanostructures will kill the bacterial cells. Other surface characteristics such as low surface energy, wettability and air entrapment may affect the ability of bacterial cell to come in direct contact with the nanostructures, thereby keeping the surfaces always free of bacteria [61]. In a recent study, extreme antibiofouling activity was demonstrated on superhydrophobic Al substrates [62]. This was due to entrapped air, the low surface energy and nanotopography of the fabricated Al substrates. In order to produce nanostructures, there are alternative fabrication strategies such as UV imprint lithography, colloidal self-assembly, electron beam lithography, anodizing, micromachining and reactive ion etching. However, such surfaces are miniaturized, expensive, time consuming and difficult to fabricate. In contrast, here we have demonstrated a facile, one-step, low cost strategy to produce a random multi-scale roughness on Al surfaces at large scale. With widespread use of Al alloys in several industries such a surface engineering strategy is expected to impact several fields to minimize biofilm formation. Specifically such surfaces can be used to prepare aseptic surfaces in public spaces including furniture, air flow ducts, surgical equipment, etc., in critical hospital environments such as operation theaters, intensive care unit and neonatal units among others.

Toward rationally designing bactericidal surfaces, this study offers considerations for the range of roughness values for fabrication of micro- and nanostructured materials. Note that the combination of the range for the parameters reported herein is not a unique combination for preparing for a bactericidal surface. In other words, a bactericidal surface may be generated with roughness values lying outside the range prescribed here but our observations suggest that a surface with roughness values described above will most likely exhibit high bactericidal efficiency. The effect of other roughness parameters not included here could affect the bacterial adhesion and needs further investigation. A comparative analysis of patterned and random nanotopographies affecting the bactericidal activity could enhance the current state of understanding in this field.

Aside from surface roughness, the other critical parameters that should be studied and correlated with the bactericidal activity include surface wettability and surface energy [56], [59], [63]. Not all hydrophobic surfaces will resist the cells whereas not all hydrophilic surfaces will attract the cells [39], [64], [65], [66], [67]. Therefore, more investigations are warranted to understand the dependence of bactericidal activity on surface energetics of such topographical features. Also, to better understand the adhesion behavior, bacterial strains of various shapes and membrane compositions may also be studied.

## 5 Conclusion

A simple etching method to generate multi-scale roughness on the surfaces of Al alloys was developed. The etching step is rapidly performed on large sized substrates. The resulting surface is highly bactericidal and the surface displays antibiofouling property against rod-shaped and coccoid shaped bacterial cells. The antibacterial activity is ascribed to the surface topography. It is proposed that a combination of various roughness parameters with similar values will be able to generate a highly bactericidal surface. Particularly effective values of surface parameters for lysing a majority of the attached bacterial cells on the surfaces were identified and could form the basis for engineering antibacterial surfaces. The multi-scale topography was also found to be effective against several drug resistant strains isolated from hospital environments. Such etched Al surfaces with excellent antibacterial activity are expected to benefit several industries in particular in hospital environments to minimize spread of nosocomial infections.

## Appendix A Supplementary data

Supplementary material Antimicrobial susceptibility of the hospital strains; 3D AFM micrographs etched cp Al samples; Optical profilometer images of etched Al 1200 and Al 5052 surfaces; Photographs and SEM images of large-scale etched samples; XPS profiles of the etched samples; XRD patterns of etched surfaces; Water contact angle of etched alloy surfaces; Bacterial viability on the etched cp Al surfaces; SEM and bacterial viability on etched Al 1200 and Al 5052 alloy surfaces; non-parametric regression analysis of the effect of surface topography on bactericidal activity; AFM images of all the etched surfaces. Image 1
