# Supplementary material for: Multi-scale surface topography to minimize adherence and viability of nosocomial drug-resistant bacteria
Source: Mater Des. 2018 Feb 15;140:332–44. doi: 10.1016/j.matdes.2017.11.074 (PMC5788004; doi:10.1016/j.matdes.2017.11.074)
Supplement: Supplementary material — Antimicrobial susceptibility of the hospital strains; 3D AFM micrographs etched cp Al samples; Optical profilometer images of etched Al 1200 and Al 5052 surfaces; Photographs and SEM images of large-scale etched samples; XPS profiles of the etched samples; XRD patterns of etched surfaces; Water contact angle of etched alloy surfaces; Bacterial viability on the etched cp Al surfaces; SEM and bacterial viability on etched Al 1200 and Al 5052 alloy surfaces; non-parametric regression analysis of the effect of surface topography on bactericidal activity; AFM images of all the etched surfaces. [file mmc1.pdf]

<u>Supporting Information for</u>

Multi-scale Surface Topography to Minimize Adherence and Viability of Nosocomial Drug-Resistant Bacteria

Jafar Hasan[1], Shubham Jain[1], Rinsha Padmarajan[1], Swathi Purighalla[2],

Vasan K. Sambandamurthy[2], Kaushik Chatterjee[1]*

[1]Department of Materials Engineering

Indian Institute of Science, Bangalore 560012 India

[2]Mazumdar Shaw Centre for Translational Research

NH Health City, Bangalore 560099 India

* *author to whom all correspondence should be addressed:*

Email: kchatterjee@iisc.ac.in; Tel: +91-80-22933408

Supporting Information Text

Antimicrobial susceptibility of the hospital strains

In vitro susceptibility testing was performed on all isolates and interpreted using the Phoenix™ (BD Diagnostic Systems) for the following antibiotics: ceftazidime, amikacin, tobramycin, ciprofloxacin, gentamicin, ticarcillin-clavulanic acid, tazobactam-piperacillin, colistin, cefepime, levofloxacin, meropenem, ceftriaxone, ampicillin, aztreonam, ertapenem, imipenem, cefoxitin, trimethoprim-sulfamethoxazole, amoxicillin-clavulanic acid and tigecycline. For S. aureus MIC was tested using the following antibiotics: amoxicillin, cefazolin, cefoxitin, chloramphenicol, daptomycin, gentamicin, linezolid, rifampicin, vancomycin. MIC values obtained by the above methods were categorized according to NCCLS breakpoints, as susceptible (S), intermediate (I), or resistant (R). *Escherichia coli* ATCC 39922 and *Pseudomonas aeruginosa* ATCC 27853 were used as quality control strains for the antimicrobial susceptibility test.

Table S1. Information of the clinical microorganisms.

ISOLATE	IDENTIFICATION	ISOLATED FROM	SENSITIVITY PROFILE
EC1	*E. coli*	Soap dispenser	ceftazidime, amikacin, tobramycin, ciprofloxacin, gentamicin, ticarcillin-clavulanic acid, tazobactam-piperacillin, colistin, cefepime, levofloxacin, meropenem, ceftriaxone, ampicillin, aztreonam, ertapenem, imipenem, cefoxitin, trimethoprim-sulfamethoxazole, amoxicillin-clavulanic acid and tigecycline
EC2252	*P. aeruginosa*	Sink	amikacin, aztreonam, cefepime, ceftazidime, ciprofloxacin, colistin, gentamicin, imipenem, meropenem, ertapenem, tazobactam-piperacillin, ticarcillin-clavulanic acid, tobramycin
EC2254	*K. pneumoniae*	Sink	amikacin, cefoxitin, colistin, ertapenem, gentamicin, imipenem, meropenem, tigecycline
B11053	*E. coli*	Patient	amikacin, gentamicin, colistin

Table S2. Information of the two aluminum alloys used in the study.

	**Aluminum 1200**	**Aluminum 5052**
Composition	Al, Si + Fe, Zn, Cu, Ti, Mn	Al, Mg and Cr
Tensile strength	70-105 MPa	210 MPa
Melting Point	660 °C	605 °C
Elasticity modulus	69 GPa	70 GPa
Thermal expansion coefficient	24×10^{-6} /K	23.7×10^{-6} /K
Applications	Pressure vessels, kitchenware, construction, roofing, ship-buildings	Kitchen cabinets, crates, aircraft and hydraulic tubes, containers, fencing

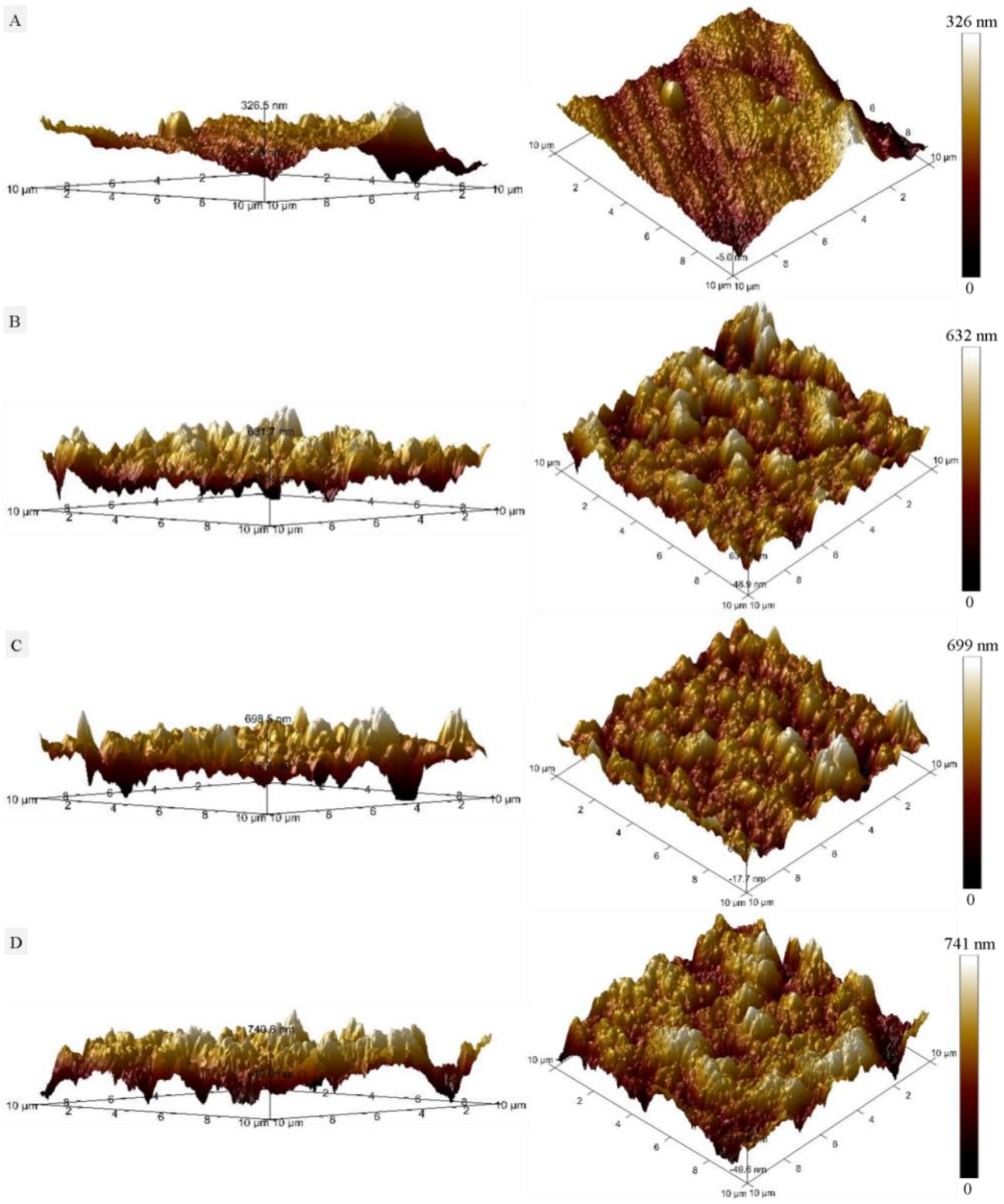

Figure S1. 3D AFM micrographs of (A) un-etched, (B) 10 min, (C) 30 min and (D) 60 min etched cp Al samples.

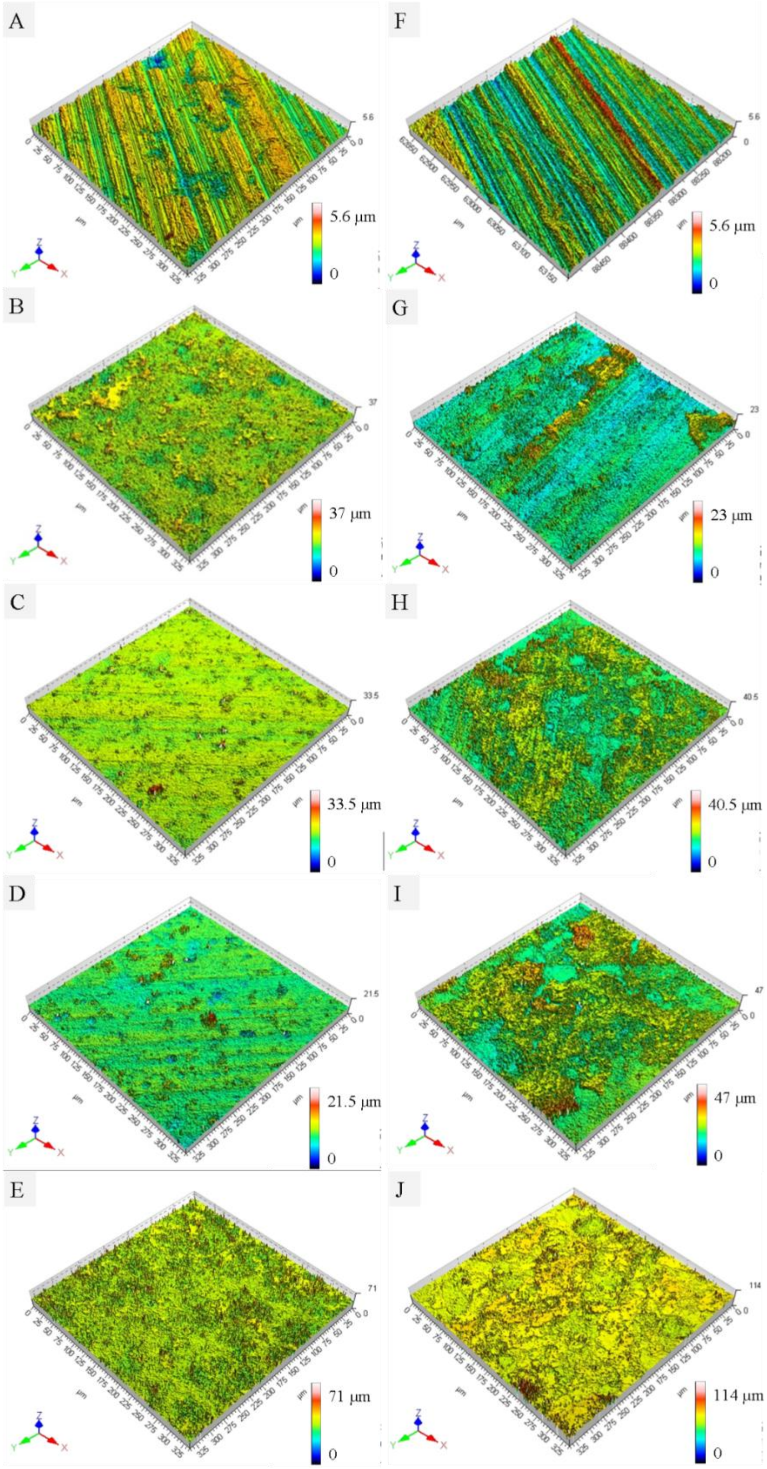

Figure S2. Three dimensional optical profilometer images of (A, F) un-etched, (B, G) 10 min, (C, H) 30 min, (D, I) 60 min and (E, J) 180 min etched Al 1200 and Al 5052 alloy surfaces respectively.

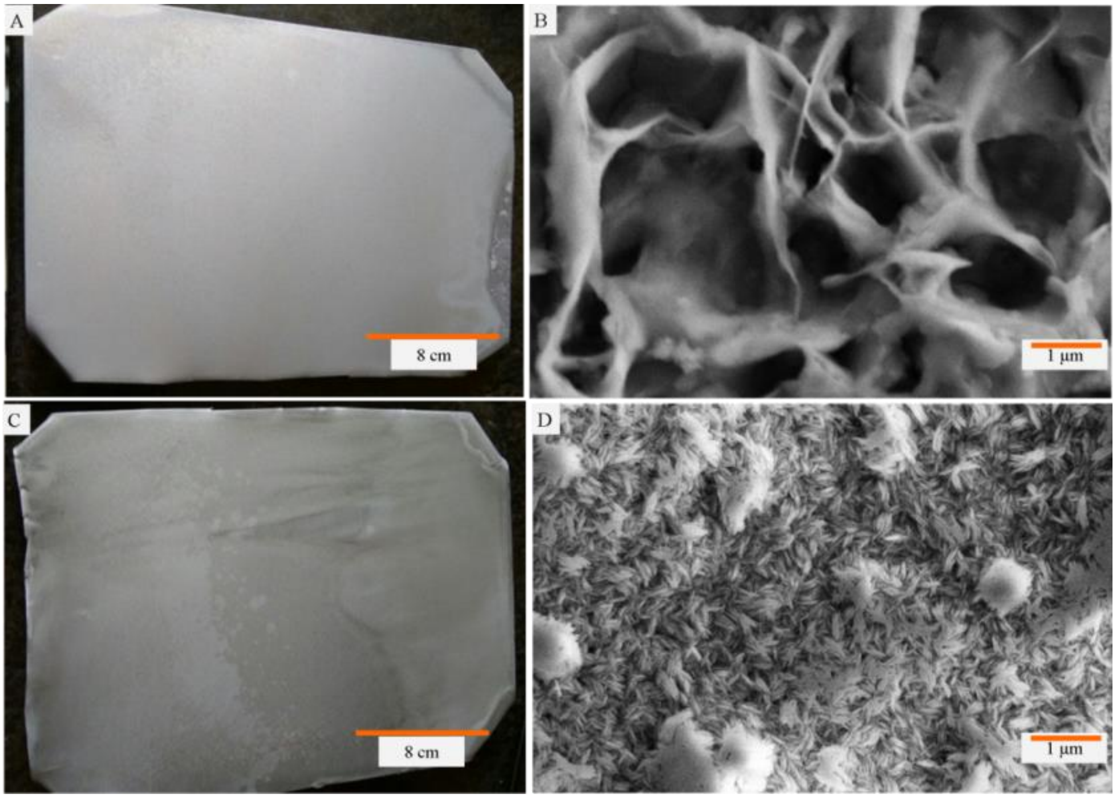

Figure S3. Photographs (A, C) and SEM images (B, D) of large-scale samples of 30 minute etched Al 1200 and Al 5052 alloys, respectively, reveal structures that resemble the surface features seen on the smaller test samples.

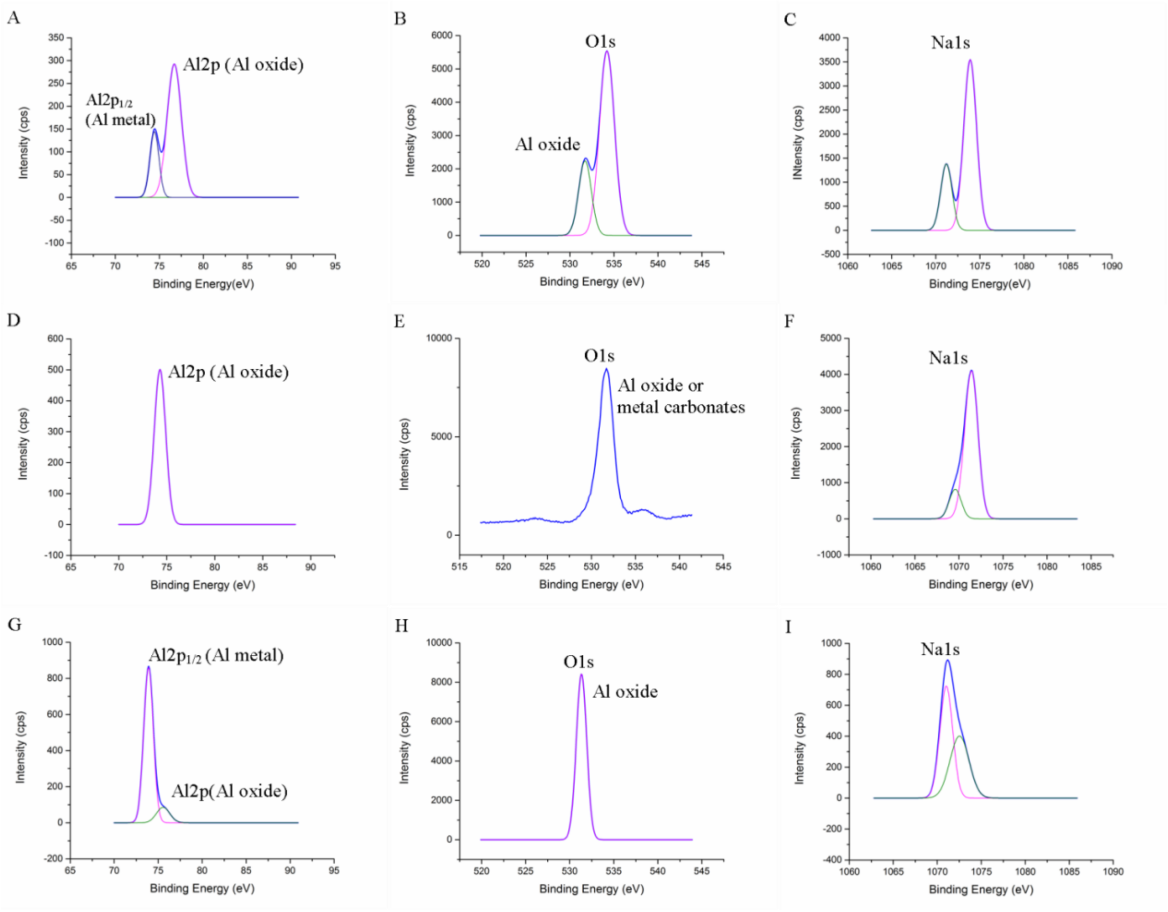

Figure S4. X-ray photoelectron spectroscopy of Al2p, O1s and Na1s peaks of the etched samples of (A, B, C) cp Al, (D, E, F) Al 1200 alloy and (G, H, I) Al 5052 alloy, respectively.

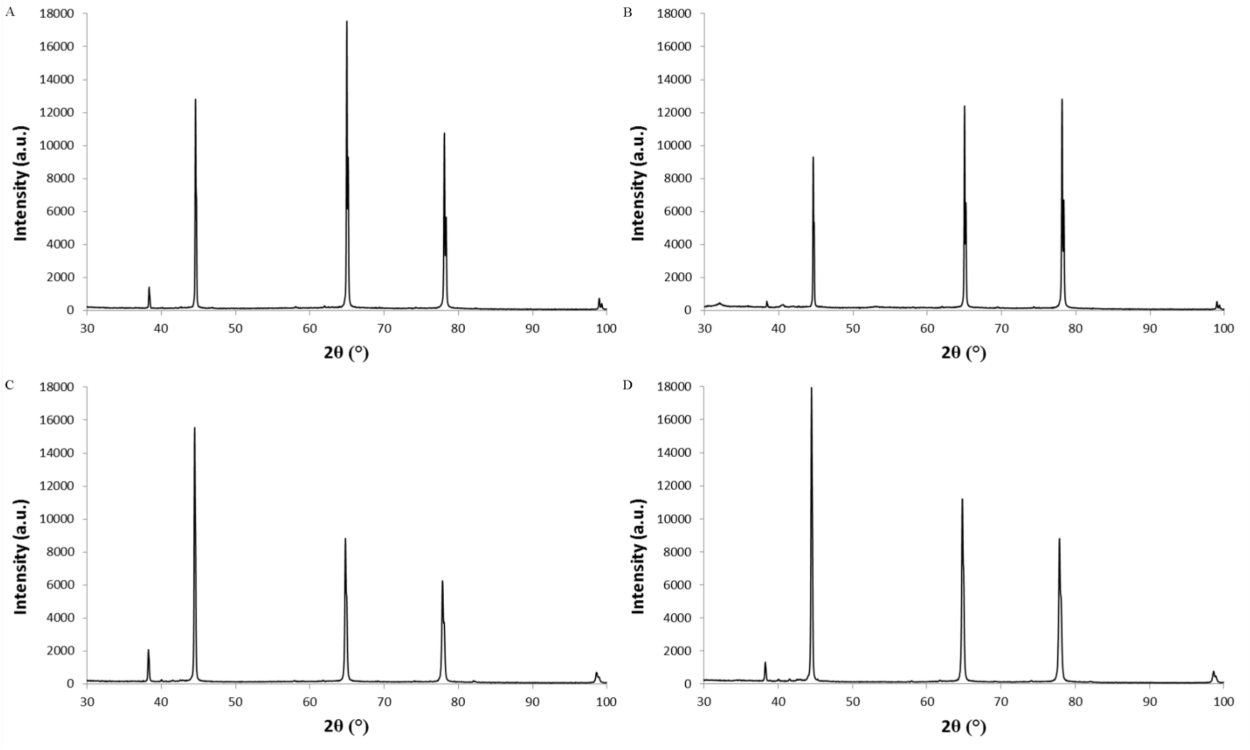

Figure S5. XRD patterns of (A) control Al 1200 alloy, (B) 10 min etched Al 1200 alloy, (C) control Al 5052 alloy and (D) 10 min etched Al 5052 alloy.

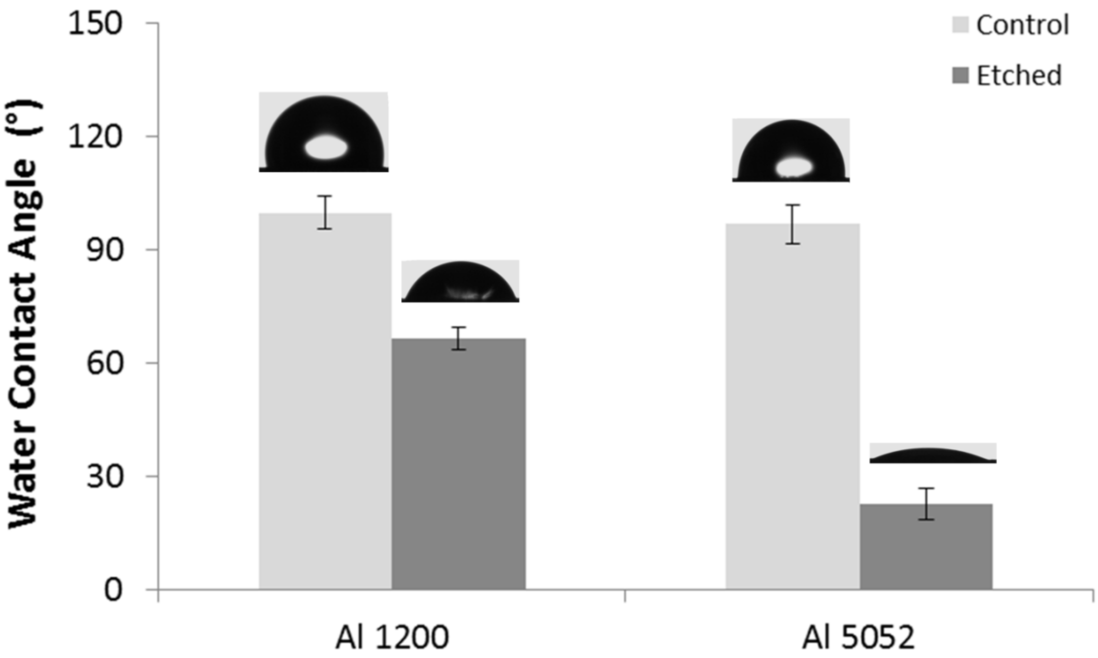

Figure S6. Contact angle values of the water droplet on the control and 10 minute etched alloy surfaces.

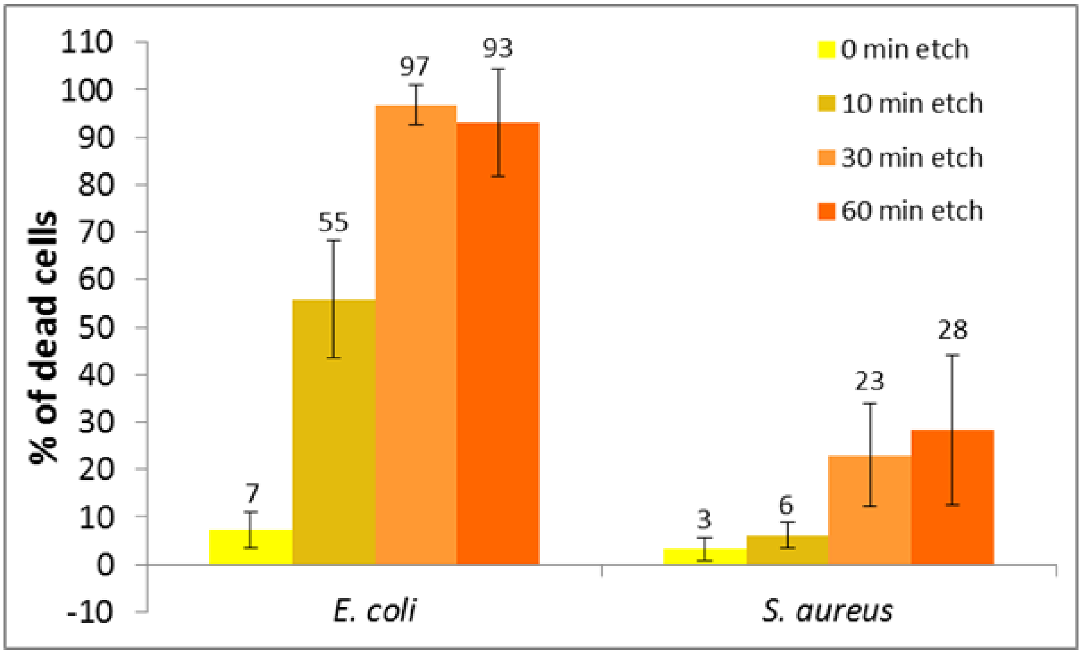

Figure S7. Quantification of the non-viable bacterial cells from Live/Dead micrographs on the cp Al surfaces after 4 hours of incubation.

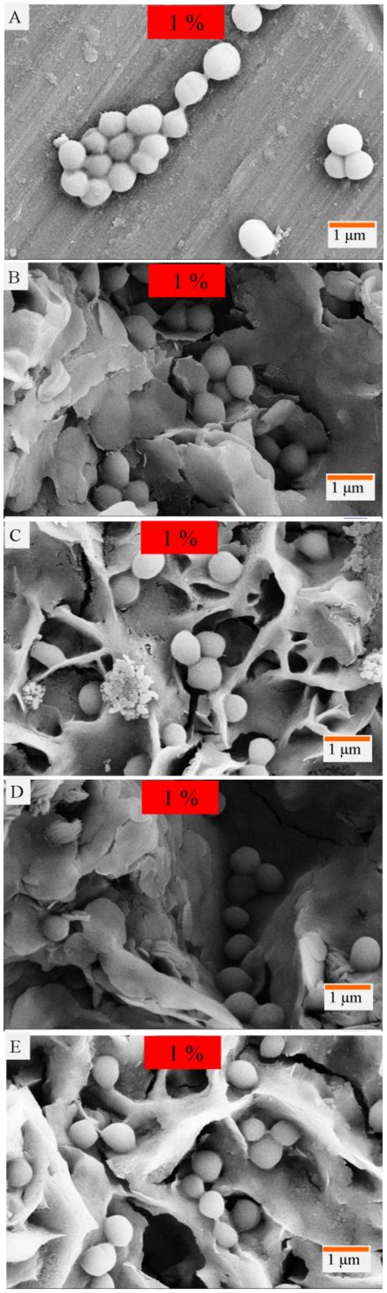

Figure S8. SEM of bacterial attachment for 4 hours of *S. aureus* cells on (A) un-etched, (B) 10 min, (C) 30 min, (D) 60 min and (E) 180 min etched Al 1200 alloy surfaces. The percentage of attached dead cells on the surfaces is also presented.

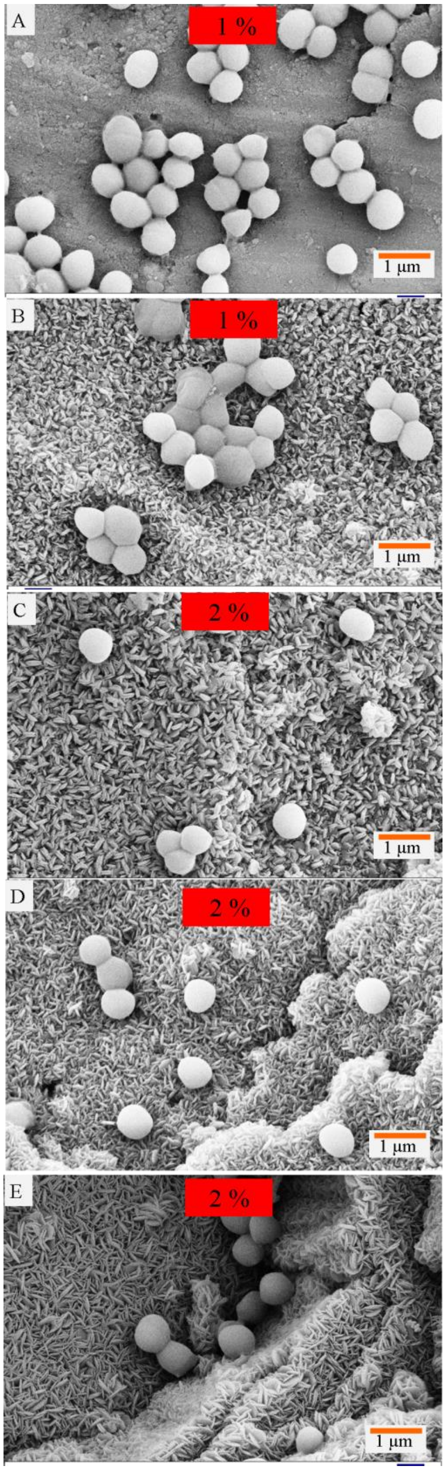

Figure S9. SEM and fluorescent images of bacterial attachment for 4 hours of *S. aureus* cells on (A, B) un-etched, (C, D) 10 min, (E, F) 30 min, (G, H) 60 min and (I, J) 180 min etched Al 5052 alloy surfaces. The percentage of attached dead cells on the surfaces is also presented.

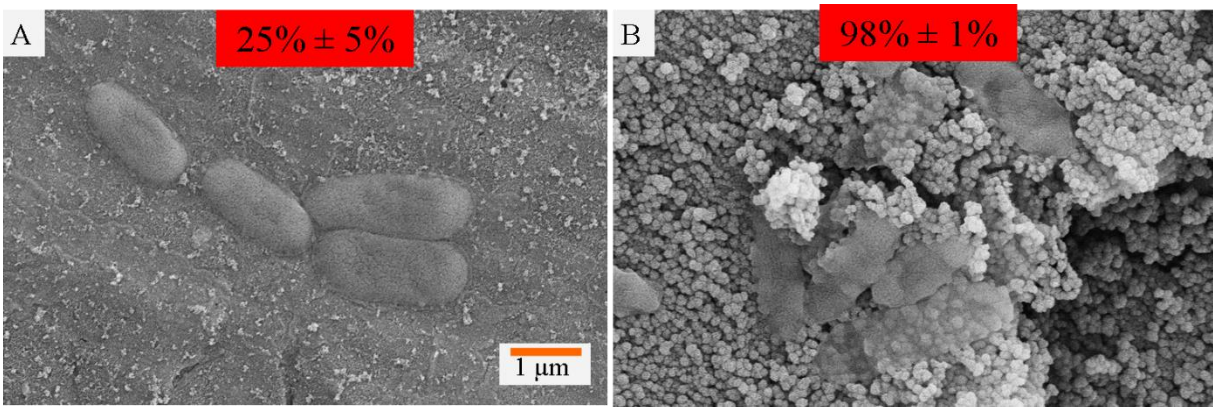

Figure S10. SEM of bacterial attachment for 4 hours of *E. coli* cells on 0.1 M of NaOH etched Al 5052 alloy surfaces for (A) 10 min and (B) 180 min etching time. The percentage of attached dead cells on the surfaces is also presented.

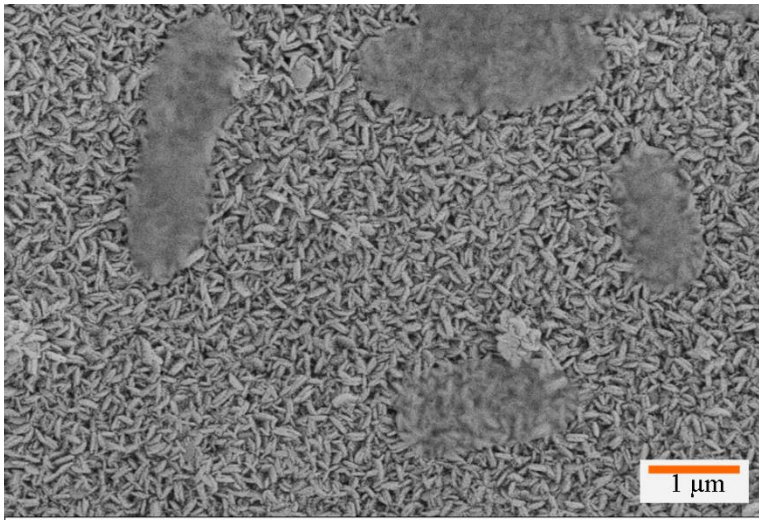

Figure S11. (A) SEM image of bacterial attachment of *E. coli* cells for 4 hours on gold coated Al 5052 alloy surfaces etched with 1 M NaOH solution for 10 minutes.

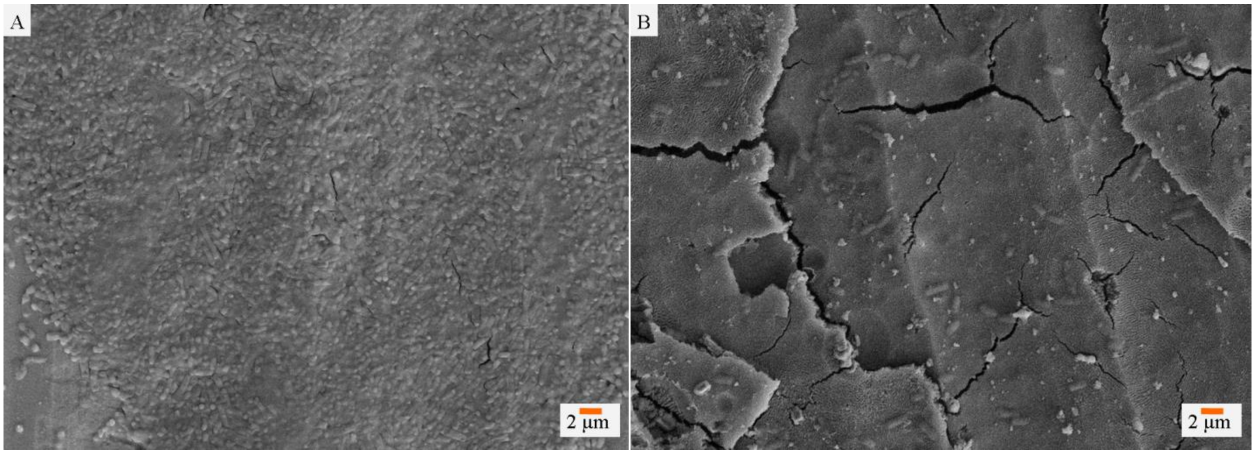

Figure S12. SEM of *E. coli* cells attached for 2 days on the (A) control and (B) 10 minute etched Al 5052 alloy. Biofilm is clearly seen on the control surface whereas the etched surface kills as well as repels the bacterial cells.

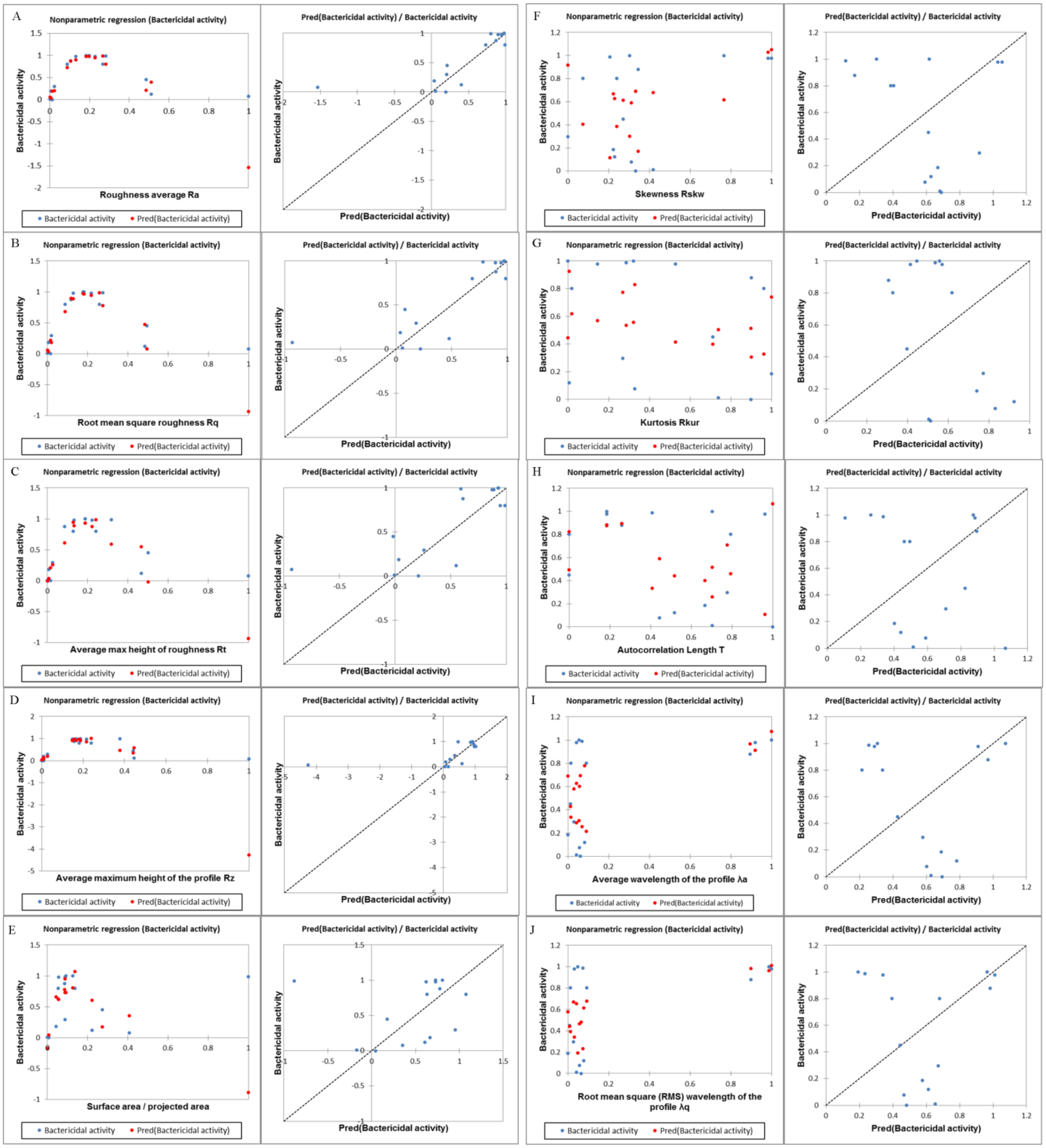

Figure S13. (A-J) Changes in bactericidal activity as a function of different roughness parameters plotted using non-parametric regression analysis of LOWESS method. The corresponding plots (right) demonstrate the relationship between the observed data and predictions as a function of the respective parameter. The model predicts that there is some correlation between some roughness

parameters (A-E) and bactericidal activity, whereas other roughness parameters (F-J) and bactericidal activity are not closely related. For each observation of the input data, a predicted value by the model is also seen. The dependent variable is the bactericidal activity whereas the roughness parameters are taken as independent variables. The roughness and bactericidal percentage values are expressed as relative to their original values such that every value is normalized between 0 and 1.

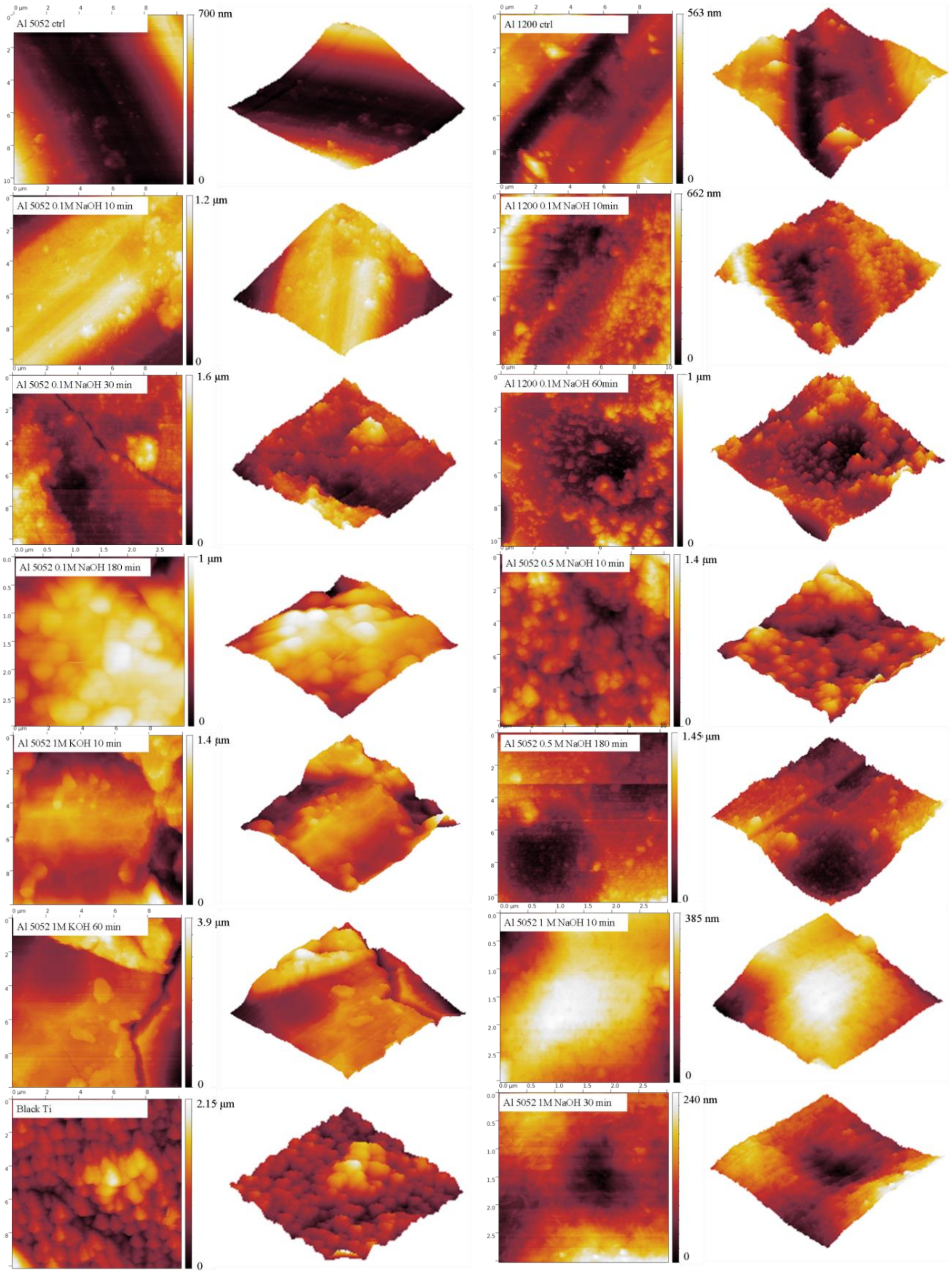

Figure S14. 2D AFM and corresponding 3D AFM images of all the surfaces tested in the study with scanned areas of 10 µm × 10 µm (corresponding to Table 1).
